# Exploring the Solubility Limits of Edaravone in Neat Solvents and Binary Mixtures: Experimental and Machine Learning Study

**DOI:** 10.3390/molecules28196877

**Published:** 2023-09-29

**Authors:** Maciej Przybyłek, Tomasz Jeliński, Magdalena Mianowana, Kinga Misiak, Piotr Cysewski

**Affiliations:** Department of Physical Chemistry, Pharmacy Faculty, Collegium Medicum of Bydgoszcz, Nicolaus Copernicus University in Toruń, Kurpińskiego 5, 85-096 Bydgoszcz, Poland; tomasz.jelinski@cm.umk.pl (T.J.); 300804@stud.umk.pl (M.M.); 300805@stud.umk.pl (K.M.)

**Keywords:** edaravone, solubility, green solvents, deep learning, COSMO-RS, learning curve analysis, hyperparameter tuning

## Abstract

This study explores the edaravone solubility space encompassing both neat and binary dissolution media. Efforts were made to reveal the inherent concentration limits of common pure and mixed solvents. For this purpose, the published solubility data of the title drug were scrupulously inspected and cured, which made the dataset consistent and coherent. However, the lack of some important types of solvents in the collection called for an extension of the available pool of edaravone solubility data. Hence, new measurements were performed to collect edaravone solubility values in polar non-protic and diprotic media. Such an extended set of data was used in the machine learning process for tuning the parameters of regressor models and formulating the ensemble for predicting new data. In both phases, namely the model training and ensemble formulation, close attention was paid not only to minimizing the deviation of computed values from the experimental ones but also to ensuring high predictive power and accurate solubility computations for new systems. Furthermore, the environmental friendliness characteristics determined based on the common green solvent selection criteria, were included in the analysis. Our applied protocol led to the conclusion that the solubility space defined by ordinary solvents is limited, and it is unlikely to find solvents that are better suited for edaravone dissolution than those described in this manuscript. The theoretical framework presented in this study provides a precise guideline for conducting experiments, as well as saving time and resources in the pursuit of new findings.

## 1. Introduction

Edaravone (5-methyl-2-phenyl-4H-pyrazol-3-one, EDA), as an active pharmaceutical ingredient (API), is used for the treatment of ischemic stroke [1,2] and amyotrophic lateral sclerosis (ALS) [1,3]. These neuroprotective actions arise from the fact that edaravone, being a free radical scavenger, has serious anti-oxidant activity [4,5]. There is, however, an important limitation in the performance of edaravone; namely, its poor aqueous solubility, documented by its categorization as a Class IV drug in the Biopharmaceutics Classification System (BCS).

The solubility of chemical compounds plays a vital role in both theoretical and practical applications [6,7]. It is widely recognized that solubility has a significant impact on bioavailability [8,9]. Hence, its enhancement remains of paramount significance in drug design and has been the subject of extensive research [10,11,12,13,14].

Another interesting domain in which the solubility of pharmaceuticals and consequently solvent selection play an important role is bioassay optimization [15,16,17,18]. It is worth noting that in the case of biological activity assessment, water–organic solvents typically containing DMSO are very often used. While water is, of course, the medium most closely resembling physiological conditions, the necessity to utilize water–organic systems arises due to the limited solubility of many biologically active substances. In this context, it should be emphasized that the solubility of pharmaceuticals, including the title compound, in aqueous–organic and organic–organic solvent mixtures has been extensively studied both experimentally and theoretically [6,19,20,21,22,23,24,25,26,27,28,29,30,31,32].

It is also worth highlighting that, apart from bioavailability and biological activity determination, solubility is of crucial importance in pharmaceutical technology, particularly concerning the selection of solvents for drug manufacturing processes [33,34]. In fact, the significance of solvents is substantial, as they account for as much as 90% of the total volume of chemicals used in the drug manufacturing process [35]. Their versatile applications encompass the synthesis of active pharmaceutical ingredients (APIs) [36,37,38], as well as separation and purification techniques (crystallization, extraction) [33,38,39,40]. Importantly, as the pharmaceutical industry places a growing focus on environmentally friendly technologies, the necessity of exploring “green” alternatives to traditional organic solvents has arisen [41,42,43,44].

In the case of edaravone, its solubility was studied in aqueous binary solvents, mixed organic solvents, as well as neat solvents, including water [21,26,45]. Additionally our research group contributed to these efforts by studying the solubility of edaravone in aqueous solutions of deep eutectic solvents [45]. The selection of an appropriate solvent in order to overcome the limited solubility of a particular API can be a tedious and difficult task. The number of experiments that can be performed is limited not only by such factors as laboratory time and financial aspects but also by the ongoing trend of restricting the usage of chemicals in the framework of green chemistry. It seems, therefore, that a screening stage, utilizing different computational methods, is necessary before starting actual experiments [46,47,48,49]. Machine learning can offer valuable help in this process. Therefore, the application of machine learning for the determination of the solubility limits of pharmaceuticals deserves special attention [50].

The main objective of this work is to demonstrate the effectiveness of the machine learning approach for exploring the extended solvent space of edaravone with the aim of screening for new solvents with experimental validation.

## 2. Results and Discussion

### 2.1. Solubility Dataset

**Figure 1:**
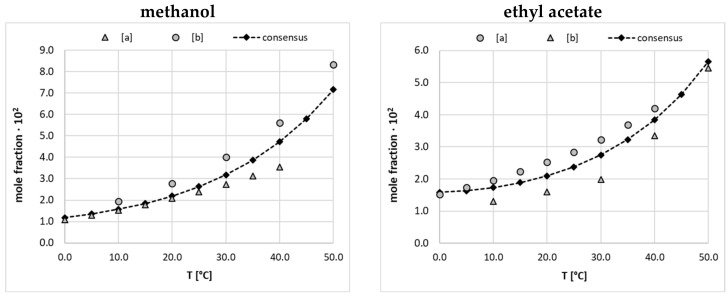
Results of data curation of EDA solubility in neat methanol and ethyl acetate using values measured by [a] Li et al. [21] and [b] Qiu et al. [26]. The consensus lines characterize fitting to the van’t Hoff equation and black diamonds define solubility data included in the final dataset.

The dataset characterizing edaravone solubility is an accumulation of values collected from available literature sources augmented with a series of new measurements reported in this paper, which are summarized in Supplementary Section S1. There are three accounts documenting the temperature-dependent solubility of the titled compound in fifteen neat solvents including alcohols, esters, some aprotic solvents [21,26], and water [45]. Moreover, nine binary solvent mixtures were used for EDA dissolution measurements [21] at a range of temperatures with a variety of binary compositions. It might seem that such a collection is extended enough for machine learning purposes; however, a closer inspection reveals three fundamental problems, which are addressed in this study. First of all, one can notice serious divergences in the reported solubility values for some systems. This inconsistency prohibits the direct use of such a collection for the training of models due to the inherent noise in the dataset, which is a frequently occurring problem intrinsic to diverse measurement protocols. Hence, such methodological divergences require careful consideration [51] prior to model formulation. Secondly, the solubility space is not represented uniformly due to different numbers of measurements for dissimilar temperature ranges and concentrations of solvent mixtures, which might overrepresent those systems studied more extensively in the dataset. Finally, the main focus in solubility determination was narrowed to polar protic solvents, with very limited representation of non-protic or diprotic solvents. To address the first two issues, the final collection was cured using commonly accepted model equations by fitting their parameters based on experimental mole fractions. Here, the three-parameter van’t Hoff and Jouyban–Acree equations were used for neat and binary solvents, respectively. In addition, temperature standardization was performed for a uniform representation of solubility in the final dataset. In Figure 1, there are examples of the results of the data curation for two selected neat solvents, which have been found to be the most problematic. The complete list of experimental solubility data is provided in the Supporting Materials (see Section S2). As can be directly inferred from the plots presented in Figure 1, incongruences appear not only in the solubility values but also in their temperature trends. The highest discrepancies were observed for EDA solubility measured in ethyl acetate. Since only an arbitrary decision would allow the rejection of either of the measurement series, the final solubility dataset was constructed based on the predictions of the van’t Hoff equation, parametrized using all available experimental data. Moreover, temperature normalization was adopted by accepting data between 0 °C and 50 °C with 5 °C intervals. Hence, points marked with black diamonds constitute the final solubility dataset. Fortunately, the majority of saturated EDA systems studied suffered experimentally from much smaller deviations, as detailed in the Supplementary Materials (see Section S2.1), and the ones presented in Figure 1 show the worst extremes.

**Figure 2:**
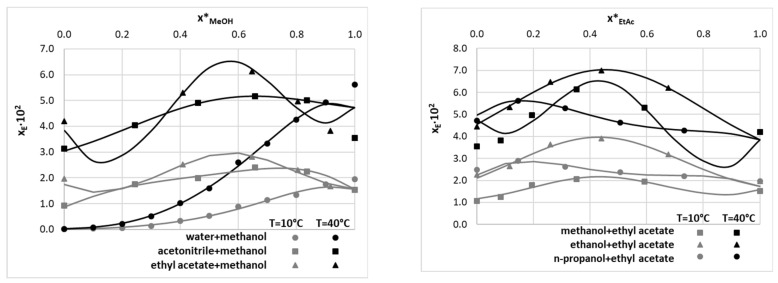
Results of data curation of EDA solubility in exemplary binary solvents using values measured by Li et al. [21]. The consensus lines characterize fitting to the Jouyban–Acree equation. The gray and black colors of markers and lines are used to distinguish lower from higher temperatures. The x_E_, x*_MeOH_, and x*_EtAc_ symbols denote the mole fraction solubility of EDA, the mole fraction of methanol, and the mole fraction of ethyl acetate in solute-free solutions, respectively.

However, these two systems are very important from the perspective of solubility data curation in binary mixtures, as frequently selected components of such complex solvents. Indeed, in Figure 2, exemplary plots are presented characterizing EDA solubility measured at *T* = 0 °C and 40 °C in binary solvents comprising either of the two solvents discussed above. All fitting results regarding binary solvents are presented in Supplementary Section S2.2.

Two very important conclusions can be drawn from the content of Figure 2. First of all, the JA model performs very well for such systems for which the set of solvent compositions is extended enough. Fortunately, this is the case for the majority of systems, except for the ethyl acetate–methanol binary mixture. In this case, the fitting results in precise back-computed solubility data but probably fails for other compositions not studied experimentally. Such serious non-monotonous behavior of the solubility line is rather unexpected, and the observed trends should be attributed to the high flexibility of the JA equation rather than to the physical phenomenon. The second important aspect is related to the abovementioned incongruences in neat solvent solubility, especially pronounced at elevated temperatures. The reason for this is that after standardizing the solubility data for pure solvents, the values obtained were also used for binary mixtures, which affected some predictions of the JA model. However, this is not an issue from the perspective of dataset curation, provided that only experimentally studied compositions are included. Indeed, the corresponding back-computed values perfectly match the experimental ones, preserving the congruency of solubility determined in both binary mixtures and neat solvents. Hence, in the final dataset, the solubility values for binary mixtures were included as computed from the JA model without applying concentration standardization.

All systems are characterized in Supporting Materials by providing graphical representations of the solubility trends, the values of the fitted parameters of applied models, and the elementary statistical measures quantifying the fitting accuracy.

### 2.2. Extension of EDA Solubility Space with Neat Solvents

The solubility dataset obtained after data curation and standardization still needs some attention due to the limited diversity of the included solvents. In order to extend the solubility space, new EDA solubility measurements were performed in a more diverse set of solvents. For this purpose, several neat solvents of the polar aprotic type were included, namely diglyme (DIG), triglyme (TIG), tetraglyme (TEG), dimethyl sulfoxide (DMSO), 1-methyl-2-pyrrolidone, (NMP), and 4-formylmorpholine (4FM). Moreover, polar diprotic solvents were taken into account by the inclusion of 2,4-dimethylphenol (DMP), 1,2-propanediol (PG), diethylene glycol (DG), triethylene glycol (TG), and 1,3-butanediol (BG). Detailed results of the measurements carried out for new systems can be found in Supplementary Materials Tables S1 and S2. Additionally, since the solvent can affect the crystalline form of the solid, and hence its thermodynamic properties, the solid residues obtained after the solubility determination procedure were analyzed using DSC and FTIR-ATR techniques (see Supplementary Figure S1). The absence of significant differences between the thermograms and spectra recorded for the precipitates and pure EDA, such as new phase transition peaks or absorption band shifts related to new hydrogen bond formation, suggests that no polymorphic or pseudo-polymorphic transformation occurs under the applied experimental conditions.

**Figure 3:**
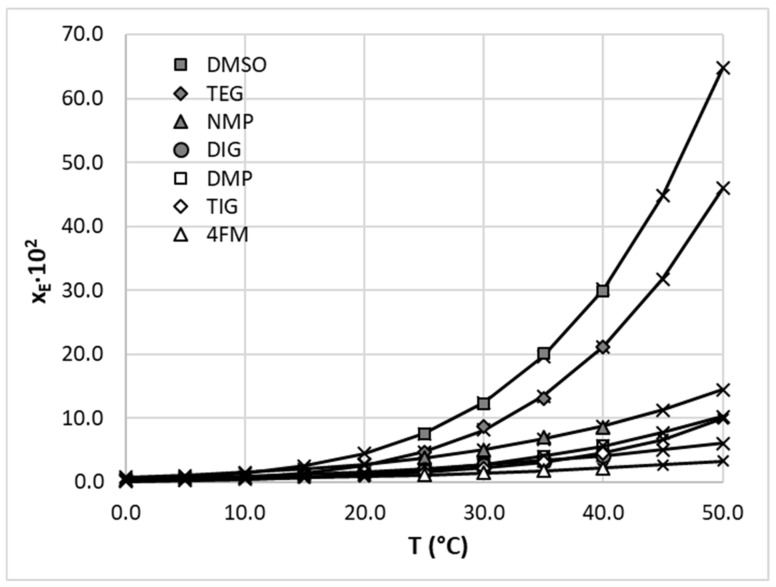
Graphical representation of mole fraction solubility of edaravone in selected polar aprotic solvents. Gray and open symbols represent measured values and crosses depict values cured using the three-parameter van’t Hoff model.

When taking into account the solubility of edaravone in neat polar aprotic solvents, it can be concluded that DMSO offers the highest dissolution potential among the studied solvents. At 25 °C, the mole fraction solubility of EDA in this solvent is equal to *x_EDA_* = 7.57 × 10^−2^, while at 40 °C, the solubility is elevated to *x_EDA_* = 29.81 × 10^−2^. DMSO is followed by TEG in terms of effectiveness, with EDA solubility amounting to *x_EDA_* = 4.63 × 10^−2^ and *x_EDA_* = 21.21 × 10^−2^ for 25 °C and 40 °C, respectively. The solubility of edaravone in other solvents is substantially lower; however the general trend of solubility increase with raised temperatures holds for all studied cases. The results are graphically depicted in Figure 3, along with values cured using the three-parameter van’t Hoff model. In addition, a single diprotic solvent, namely DMP, is listed here.

### 2.3. Extension of EDA Solubility Space with Aqueous Binary Solvents

**Figure 4:**
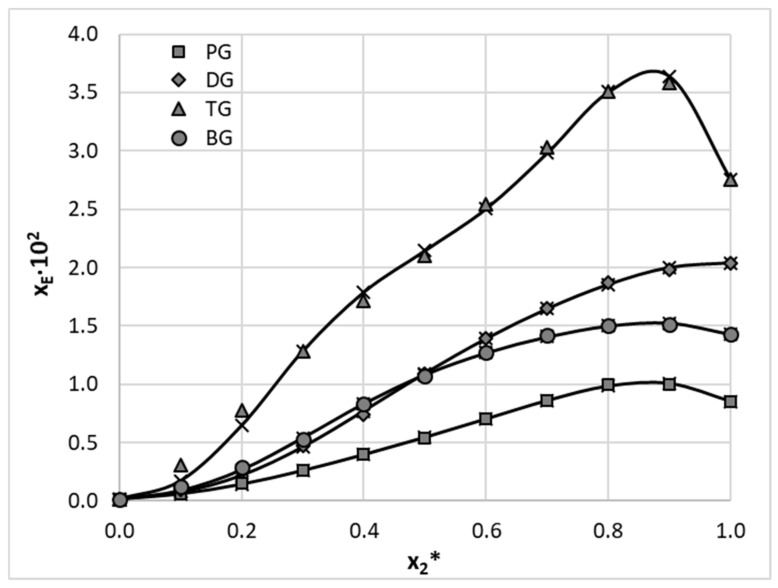
Mole fraction solubility of edaravone at 25 °C in aqueous binary mixtures of selected polar diprotic solvents. Gray symbols represent measured values and crosses depict values cured using the JA model.

Apart from using neat polar solvents, both aprotic and diprotic, additional solubility experiments were conducted for aqueous binary solvents (Supplementary Table S2). These were created by mixing four diprotic solvents with water in varying molar proportions. Quite often the addition of another solvent, for example, water, can lead to a substantial solubility increase of a particular API compared to the neat solvent, which is described as a cosolvency effect [27,52]. Triethylene glycol (TG) was responsible for the highest solubility of EDA amounting to a molar fraction of *x_EDA_* = 2.75 × 10^−2^ at 25 °C. Interestingly, the aqueous binary composition with the molar fraction of the organic solvent equal to *x*_2_* = 0.9 offered even better EDA solubility with *x_EDA_* = 3.58 × 10^−2^. In addition, for 1,3-butanediol (BG)–water and 1,2-propanediol (PG)–water mixtures, this particular composition results in higher solubility compared to pure solvents. The only exception is the binary solvent containing diethylene glycol (DG), for which no cosolvency effect was observed. The results are depicted in Figure 4, together with values cured using the Jouyban–Acree model.

### 2.4. Machine Learning Solubility Model

**Figure 5:**
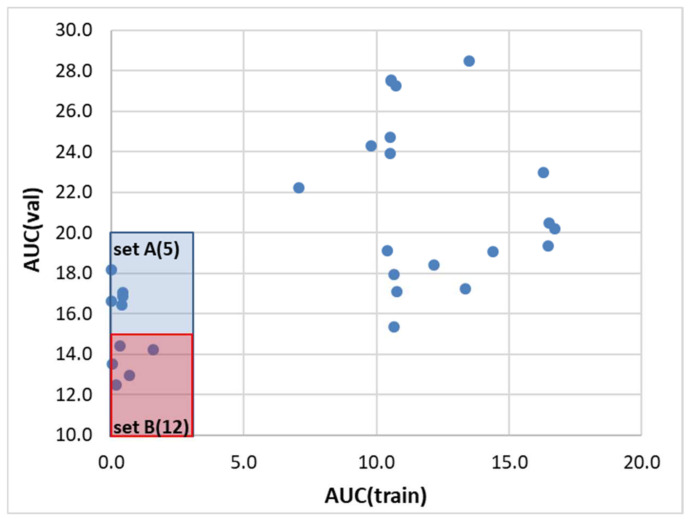
Results of regression models’ selection based on the distributions of the area under the AUC curve (blue dots) determined from learning curve analysis, loss values of test, and validation sets. Set A comprises the following five models: NuSVR, SVR, CatBoostRegressor, XGBRegressor, and HistGradientBoostingRegressor. In set B, twelve additional regressors were categorized including GaussianProcessRegressor, BaggingRegressor, RandomForestRegressor, LGBMRegressor, MLPRegressor, LassoLars, LassoLarsCV, Ridge, KNeighborsRegressor, AdaBoostRegressor, OrthogonalMatchingPursuitCV, and TransformedTargetRegressor.

The machine learning was performed by training a set of 36 regression models, which were used for the ensemble definition based on the performance and predictability potential. These parameters were assessed based on test and validation subsets not used during the training phase. It adheres to good practice to tune the parameters of the models on the training set and verify their effectiveness using a portion of the data that has not been seen before. This procedure increases the predictability of the trained models. Figure 5 shows a scatter plot of the models’ characteristics, which enables the identification of two sets of regressors with similar efficiencies. It is worth emphasizing that due to the definition of the score function used during the tuning of the models’ parameters, the results of the learning curve analysis are used as a final evaluation approach rather than mean absolute error (MAE) or the coefficient of determination (R^2^) themselves. For this purpose, the area under the curve (AUC) was determined for every regressor model with optimized parameters, for which the percentage of the sample was systematically increased from 50% up to 100% of the dataset.

Both sets of regressors comprise machine learning models commonly used for regression problems. These models have different algorithms for learning the relationship between input and output variables, as well as varying levels of complexity and hyperparameters that need to be tuned. While all models excel at handling high-dimensional input data and continuous variables, they differ in their strengths and weaknesses in terms of their ability to handle different types of data and noise levels. By grouping these models based on their performance, one can assess their effectiveness in predicting solubility and identify the most suitable model for our dataset. The first set of regressors, denoted as A, includes five models. For instance, support vector machines are often used for small datasets, while ensemble-based models such as HistGradientBoostingRegressor, CatBoostRegressor, and XGBRegressor are preferred for larger datasets. The second set of regressors, marked B, includes twelve models. Among them, GaussianProcessRegressor is known for its ability to model complex functions and handle small datasets, while ensemble-based models such as BaggingRegressor, RandomForestRegressor, and AdaBoostRegressor are often used for larger datasets.

Using a set of models, instead of relying on a single best-performing one, can offer several benefits. Firstly, it allows for the evaluation of the performance of multiple models by averaging their predictions. This takes advantage of the strengths of regressors from complementary models that can capture different aspects of the data, providing more robust predictions. Grouping models into subsets based on their predicting abilities provides additional validation of the overall performance by comparing both back computations and new predictions. The fact that the yielded mean values and standard deviations were very similar is a good prognostic for practical ensemble applications. Secondly, using a set of models can help mitigate the risk of overfitting to a particular model architecture or hyperparameters, which can be a common issue when relying on a single best model. Therefore, using an ensemble of models can provide a more comprehensive and reliable approach for predicting solubility and other regression problems. Hence, the ensemble comprising all the subsets was used for EDA solubility computations in the extended set of neat solvents and binary mixtures. The details of ensemble predictions, as well as contributions from all three subsets, are provided in the Supplementary Materials (see Excel file SM_models.xlsx). Moreover, all hyperparameters tuned for corresponding regressors are provided.

**Figure 6:**
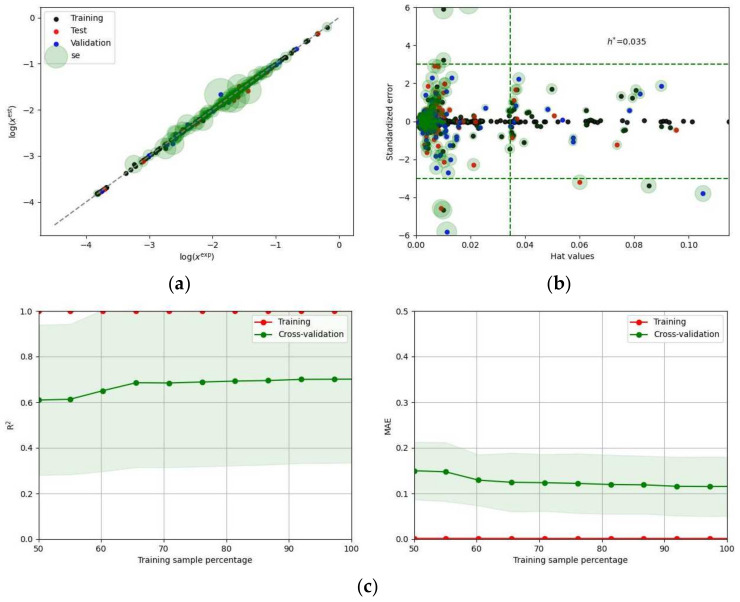
Graphical illustration of the NuSVR regression model’s performance. The panels (**a**), (**b**), and (**c**) document the correlation between computed and consensus solubility values with annotation of the standard deviation as circle’s radius, applicability domain plots, and the results of learning curve analysis concerning both R^2^ and MAE, respectively. The *x_Eest_* symbol denotes the estimated EDA solubility values.

In the caption of Figure 5, the regressor sets are ordered according to the descending values of the AUC for the validation set. It can be seen that the two best models take advantage of regression algorithms based on the support vector machine (SVM) technique. These are the NuSVR (Nu support vector regression) and SVR (support vector regression) models, and the former is generally considered more robust to outliers compared to SVR. The “nu” parameter in NuSVR controls the upper bound on the fraction of margin errors and support vectors. Adjusting this parameter enables the trade-off between the number of support vectors and the errors allowed in the training set to be controlled. SVR, on the other hand, penalizes points that lie outside the error bounds more heavily, which can make it more sensitive to outliers. The overall performance of the best model is presented in Figure 6. Similar characteristics of all other regressors included in the two subsets are presented in the Supplementary Materials (see Section S3).

### 2.5. The Solubility Space Characteristics

**Figure 7:**
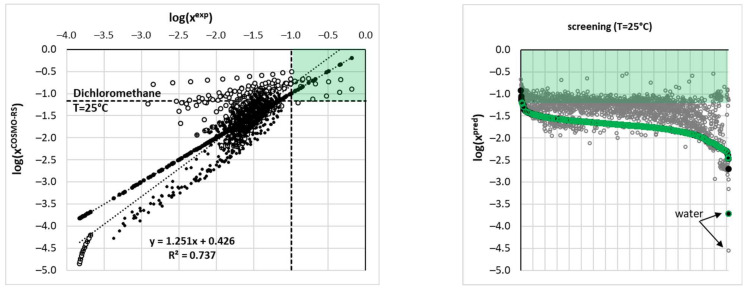
The experimentally and theoretically determined solubility values of EDA.

The main reason for ensemble model development is the extension of the solubility space for systems not studied experimentally. This cannot be carried out solely using COSMO-RS predictions, which is clearly documented in Figure 7. The left panel, presenting the correlation between computed and experimental solubility values, suggests only a qualitative accuracy of this theoretical framework. Conversely, a perfect match between estimated and measured solubility can be observed in the case of the ensemble. In the right panel, predictions made using both theoretical approaches were assorted according to increasing values of solubility derived from the machine learning model. The region marked by a green rectangle corresponds to higher EDA solubility than the one achieved in dichloromethane (which was the most effective solvent studied experimentally) at ambient conditions. Additionally, the green circles identify solvents, which are supposed to be environmentally friendly according to US Environmental Protection Agency (EPA) classification. It relies on the estimation of the so-called environmental index (EI), which in turn can be calculated using the PARIS III application [53]. This parameter includes several toxicological factors: human toxicity by inhalation (HTPInh), human toxicity by ingestion (HTPIng), aquatic toxicity (ATP), terrestrial toxicity (TTP), and physicochemical features related to ozone depletion (ODP), global warming (GWP), acid rain (AR), and photochemical oxidation (PCOP). However, when the latter factor is taken into account, EI is very high in the case of DMSO, which is commonly regarded as green. Since other physicochemical parameters, namely ODP, GWP, and AR, include all important atmospheric hazards related to the potential reactivity of the solvents, the contribution of PCOP was set to zero. For the purposes of this study, all solvents from the PARIS III collection with EI < 1.0 are regarded as green ones [28] and are marked by green circles in Figure 7. Two main conclusions can be inferred from both panels. First of all, there is very little space for solubility extensions by the application of new solvents, especially if the “greenness” criterion is imposed. Indeed, the top five ranked solvents pointed out by the ensemble model as the most suited for edaravone are collected in Table 1. It is interesting to note that all these solvents belong to the class of polar aprotic solvents. The first three seem to be almost identically effective, bearing in mind the values of the standard deviations. Hence, DMSO is supposed to fulfill the criterion of the highest solubility limit of EDA in a neat solvent. It is also unlikely that any binary mixture, except those comprising DMSO, can offer higher solubility. This conclusion cannot be so definitely stated based solely on COSMO-RS-derived solubility, as is clearly visible by the cloud of points within the green zone in Figure 7.

## 3. Materials and Methods

### 3.1. Materials

Edaravone (EDA, CAS Number: 89-25-8, MW = 174.20 g/mol) was supplied by Sigma Aldrich (Saint Louis, MO, USA) and its purity was ≥98%. The following compounds were used as solvents throughout the study: diglyme (DIG, CAS Number: 111-96-6), triglyme (TIG, CAS Number: 112-49-2), tetraglyme (TEG, CAS Number: 143-24-8), dimethyl sulfoxide (DMSO, CAS Number: 67-68-5), 1-methyl-2-pyrrolidone, (NMP, CAS Number: 872-50-4), 4-formylmorpholine (4FM, CAS Number: 4394-85-8), 2,4-dimethylphenol (DMP, CAS Number: 105-67-9), 1,2-propanediol (PG, CAS Number: 57-55-6), diethylene glycol (DG, CAS Number: 111-46-6), triethylene glycol (TG, CAS Number: 112-27-6), 1,3-butanediol (BG, CAS Number: 107-88-0), and methanol (CAS Number: 67-56-1). The above chemicals were also purchased from Sigma Aldrich and their purity was stated by the supplier as ≥ 98%. All chemicals were used as obtained without any initial procedures.

### 3.2. Solubility Measurements

To assess the solubility of EDA in various solvents, excess amounts of EDA were added to test tubes containing either a specific solvent or a binary mixture containing the organic solvent and water in different molar proportions. The saturated solutions were then placed in an Orbital Shaker Incubator ES-20/60 from Biosan (Riga, Latvia) and incubated at various temperatures for 24 h. Four temperature points, ranging from 25 °C to 40 °C with 5 °C intervals, were used for the incubation. The incubator temperature was precisely adjusted to within 0.1 degrees, with a variance of 0.5 degrees during the 24 h cycle. The samples were simultaneously mixed at 60 rev/min. Next, the samples were filtered using syringes equipped with PTFE filters with a pore size of 0.22 µm. To prevent precipitation due to temperature differences between the solutions and instruments, all test tubes, pipette tips, syringes, and filters were preheated. They were placed in the same incubator as the samples and heated to the same temperature before handling. This step was particularly crucial when dealing with elevated temperatures, as the temperature difference could be substantial. After filtration, small quantities of the obtained filtrate were diluted in test tubes containing methanol and measured spectrophotometrically. The density of each solution was measured by weighing a 1 mL volume in 10 mL volumetric flasks using an Eppendorf Reference 2 pipette (Hamburg, Germany) with a systematic error of 6 μL. The RADWAG AS 110 R2.PLUS analytical balance (Radom, Poland) with a precision of 0.1 mg was also used for this purpose. Solubility determination was conducted using the A360 spectrophotometer from AOE Instruments (Shanghai, China). Spectra were recorded in the wavelength range of 190 nm to 400 nm with a resolution of 1 nm. Methanol was used for both diluting the samples and the initial calibration of the spectrophotometer. The analytical wavelength was set at 243 nm, and the absorbance at this wavelength was used to determine the EDA concentration in the samples and subsequently calculate its mole fractions. To ensure accuracy, three separate measurements were performed, and the resulting values were averaged. The calibration curve for EDA was prepared by diluting an initial stock solution and measuring the resulting solutions’ spectrophotometric properties at decreased concentrations. The molar concentrations of the measured solutions ranged from 0.0023 to 0.023 mg/mL. The relationship between the absorbance values at 243 nm and the solution concentration was described by a linear equation A = 85.603 × C − 0.0179, with high linearity denoted by the determination coefficient R^2^ equal to 0.9993.

### 3.3. Instrumental Analysis of Solid Residues

The dried solid residues obtained after the solubility determination procedure were subjected to Fourier transform infrared spectroscopy (FTIR) and differential scanning calorimetry (DSC) measurements. For this purpose, the Perkin Elmer Spectrum Two spectrophotometer (Waltham, MA, USA) equipped with an attenuated total reflection (ATR) device and the DSC 6000 calorimeter from PerkinElmer (Waltham, MA, USA) were used. The calorimetric measurements were conducted with a heating rate of 5 K/min and a 20 mL/min nitrogen flow to create an inert atmosphere. The samples were placed in standard aluminum pans and the DSC apparatus was calibrated using indium and zinc standards prior to the measurements.

### 3.4. Solubility Data Curation

The datasets used for model development underwent curing and unification. All solubility data in neat solvents were analyzed using a simple thermodynamic model relying on the fundamental van’t Hoff equation extended for the temperature dependence of the equilibrium constant by a polynomial fit [54]. The following equation

(1) $$\ln\left(x_{E}^{cur}\right)=A+\frac{B}{T}+\frac{C}{T^{2}}$$

has three adjustable parameters, the values of which were computed by minimizing root mean square deviations (RMSD) between experimental and computed values. The collection of obtained parameter values for all analyzed systems (including literature data [21,26,45]), along with graphical illustrations, is provided in the Supplementary Materials (see Section S2, Table S3 and Figure S2).

The solubility of EDA in binary mixtures was also prone to curation. For this purpose, the Jouyban–Acree model [25,55] was used as it was proven to be able to adequately represent the spectrum of solution behavior from ideal to highly non-ideal systems [56]. This semi-empirical thermodynamic mixing model relies on a nearly ideal binary solvent/Redlich–Kister equation accounting for contributions from both two-body and three-body interactions [25]. The following adaptation was used for the purpose of this study:

(2) $$\ln\left(x_{E}^{cur}\right)=x_{1}^{*}\cdot ln\left(x_{E}^{\left(1\right)}\right)+\left(1-x_{1}^{*}\right)\cdot ln\left(x_{E}^{\left(2\right)}\right)+x_{1}^{*}\cdot \left(1-x_{1}^{*}\right)\cdot \overset{2}{\underset{i=0}{\sum }}J_{i}^{}\cdot {\left(2x_{1}^{*}-1\right)}^{i}$$

where *J*_0_, *J*_1_, and *J*_2_ are adjustable parameters and $x_{1}^{*}$ represents the mole fraction of the first solvent in the initial binary mixture. The collection of all fitted values determined in this study and obtained from the literature is provided in the Supplementary Materials (see Section S2, Table S4).

### 3.5. Model Development

For the purpose of exploring the solubility space of edaravone, an extensive search for non-linear models was performed. The full hyperparameter tuning procedure was used for 36 regression models, which were chosen based on a variety of algorithms including linear models, boosting, ensembles, nearest neighbors, neural networks, and other types of regressors. A Python code was developed specifically for this study, and the search for the optimal parameters of each model was conducted using Optuna study, a freely available Python package for hyperparameter optimization [57]. The collection of the tuned models was formulated after 5000 minimization trials using TPE (Tree-structured Parzen Estimator) as a sampler of the search algorithm. TPE is known for being computationally efficient and uses a probability density function to model the relationship between hyperparameters and performance metrics. To evaluate the performance of each regression model, a custom score function was developed, which combines multiple metrics, taking into account both the model’s accuracy and ability to generalize. This scoring function was previously discussed [28] and only a short note is provided here. In the present study, the training dataset was used for computations using Formula (3), which includes the mean squared error between the predicted and actual values of the target variable, as well as penalties on the number of positive values and outliers.

(3) $$loss_{train}=MSE_{train}^{LC,train}+\left|MSE_{train}^{LC,train}-MSE_{train}^{LC,test}\right|+\;+MSE_{train}\left(1+100\cdot N_{traint}^{pos}+10\cdot N_{train}^{out}\right)$$

where $MSE_{train}$ is the value of the mean squared error between the predicted and actual values, $N_{train}^{pos}$ is the number of positive values, and $N_{train}^{out}$ is the number of outliers, while $MSE_{train}^{LC,train}$ and $MSE_{train}^{LC,test}$ values are obtained from the learning curve analysis. The scoring function has two penalties for the number of positive values and outliers. The first penalty ensures that the predicted values are formally acceptable, as the models were trained against the values of solubility expressed as the logarithm of the mole fraction, which should always be positive. The second penalty directs the acceptance of models with as few outliers as possible, defined as values that exceed three times the standard deviation. The first two terms in Formula (3) were obtained from the learning curve analysis (LCA) of the scikit-learn 1.2.2 library [58], which provides information on the model’s performance for different training set sizes. It is worth noting that LCA utilizes cross-validation (CV), which was set to a 5-fold CV of the training dataset. The first two contributions are obtained from the learning curve analysis, which provides information on the model’s ability to generalize to new, unseen data. To perform the learning curve analysis, the sklearn.model_selection.learning_curve function from the scikit-learn library [58] was used. Due to its computational expense, only two-point computations were performed by including 50% to 100% of the total data. Overall, this approach allowed us to evaluate the performance of the models and identify the optimal training set size for each model. To assess the performance of the tuned models, a learning curve analysis (LCA) was conducted using 20-point computations. The values included in the custom loss function corresponded to the mean absolute error (MAE) values obtained at the largest training set size. By combining the two types of components, the custom loss function provided information on the model’s accuracy and ability to generalize to new, unseen data. The ensemble model (EM) was formed by selecting the subset of regression models with the lowest values for both criteria. The final predictions were obtained by averaging the predictions from the selected models. This approach allowed us to develop an ensemble of models that provided more robust and accurate predictions of solubility.

### 3.6. Molecular Descriptors

In order to develop a model for selecting effective EDA solubilizers, suitable molecular descriptors need to be selected. Selecting the molecular descriptors carrying sufficient structural information is a crucial step in the model’s development. Since the input data depend on the temperature, the quantum chemistry COSMO-RS method available in the COSMOtherm package [59] was applied [60] instead of typical QSPR/QSAR molecular features. The set of computed variables comprised intermolecular interaction descriptors, chemical potentials, activities, solubility values, gas phase properties, σ-profiles, σ-potentials, σ-moments, and other features. Notably, several previous studies revealed the high predicting power of the COSMO-RS descriptors combined with machine learning techniques [19,30,32,51,61]. In order to develop the most reliable tool for solvent screening, the sets of computed molecular descriptors were subjected to preselection according to the following inclusion criteria: (1) correlation with experimentally determined data, (2) sufficient variability, and (3) orthogonality [62].

**Figure 8:**
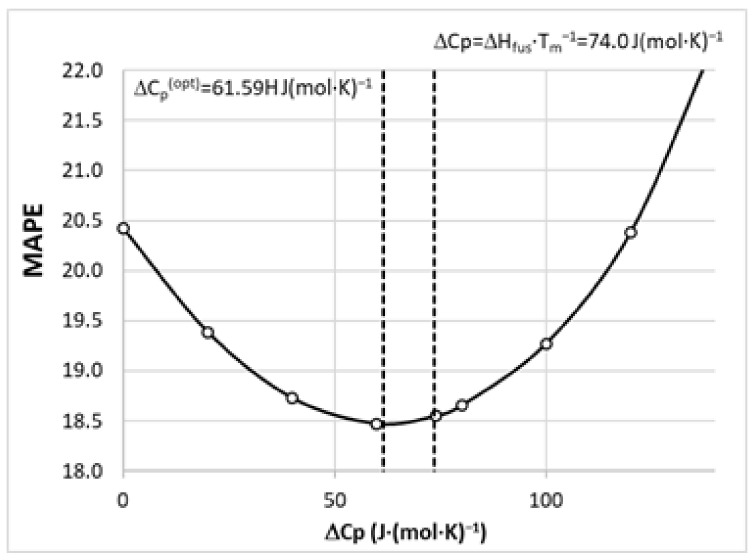
The results of optimization of the Δ*C_p_* value for solubility computations using the COSMO-RS approach.

Based on the previous studies [19,28], the COSMO-RS-computed solubility values seem to be the first-choice descriptors. Although COSMO-RS is frequently used, it is generally known as being only qualitatively accurate. There are several limitations to this approach, among which is the necessity of providing experimental values for fusion thermodynamics if solid–liquid equilibria (SLE) are the subject of interest. Luckily, for many compounds, there are available [63,64] values of melting temperatures, *T_m_*, and fusion enthalpies, Δ*H_fus_*. Indeed, for EDA, the following values are reported: *T_m_* = 127 °C [21,65] and Δ*H_fus_* = 29.61 kJ/mol [21]. However, the SLE equilibrium is generally defined by the following equation [66,67,68]:

(4) $$lna^{s}=\frac{\Delta H_{fus}^{}}{R}\cdot \left(\frac{1}{T_{m}}-\frac{1}{T}\right)+\frac{1}{R}\int_{T_{m}}^{T}\frac{\Delta C_{p}}{T}dT-\frac{1}{RT}\int_{T_{m}}^{T}\Delta C_{p}dT$$

where *R* is the gas constant, as is the solute activity in saturated systems, and Δ*Cp* stands for heat capacity change upon melting. This value is generally unavailable but seems to be important [68,69], especially for temperature ranges far from the melting point, which is surely the case for SLE measurements. Some researchers have argued [32,70] that ignoring this contribution, Δ*C_p_* = 0, introduces an acceptable estimation due to the cancelation of errors in Equation (4). On the other hand, there is evidence that Δ*C_p_* ≈ Δ*S_fus_* ≈ Δ*H_fus_* × *T_m_*^−1^ is a better choice [32,71]. To ensure as high as possible accuracy of COSMO-RS solubility estimation, preliminary computations were performed to find the value minimizing the overall mean average percentage error, MAPE, for the whole solubility dataset. Hence, several trials of solubility computations were performed for a broad range of heat capacity changes, and the resulting correlation between MAPE and the values of Δ*C_p_* is plotted in Figure 8. It is interesting to see that the performed tuning induces quite a small effect on the overall accuracy of solubility determined using COSMO-RS. The initial guess Δ*C_p_* ≈ Δ*S_fus_* ≈ Δ*H_fus_* × *T_m_*^−1^ = 74.0 J/(mol·K) is very close to the optimized value Δ*C_p_*(*opt*) = 61.59 J/(mol·K). Hence, the final set of solubilities taken for machine learning purposes corresponds to this latter value. All solubility computations were performed by allowing the SLE to be solved by COSMOtherm software (version 22.0.0) in order to avoid problems with the iterative protocol.

The second molecular descriptor selected for machine learning is the relative value of the infinite dilution activity coefficient (IDAC), $\Delta ln\left(\gamma_{ES}^{\infty }\right)$, defined as follows:

(5) $$\Delta ln\left(\gamma_{ES}^{\infty }\right)=ln\left(\gamma_{E}^{\infty }\right)-ln\left(\gamma_{S}^{\infty }\right)$$

where the *S* symbol denotes either the neat solvent or the binary mixtures. In the latter case, the value is computed simply as a sum of the neat solvent IDAC values weighted with the mole fraction composition of the mixture without the solute.

The output files generated for the purpose of IDAC computations were used for the extraction of the relative values of intermolecular interactions in the studied systems. Hence, the inclusion criteria met the following energetic terms:

(6) $$\Delta E_{ES}^{\infty }\left(int\right)=\Delta E_{E}^{\infty }\left(int\right)-\Delta E_{S}^{\infty }\left(int\right)$$

where *int* stands for the total, misfit, van der Waals, or hydrogen bonding contributions. Again, in the case of mixed solvents, the values were computed as a weighted sum of the solvents’ contributions.

The COSMO-RS theory introduced the concept of Taylor series expansion of the σ-potential:

(7) $$M_{i}^{EDA}=\int p^{EDA}\left(\sigma \right)\cdot \sigma^{i}d\sigma$$

and the resulting quantities were termed σ-moments. The zero-order σ-moment, $M_{i=0}^{BSA}$, is simply the molecular area of the EDA. The first σ-moment $M_{i=1}^{EDA}$, is the negative charge of the compound. The second σ-moment, $M_{i=2}^{EDA}$, is related to the screening charge of the system. The third and fourth σ-moments characterize σ-profile skewness and kurtosis, respectively. The COSMOtherm program (version 22.0.0) allows for computing at most the sixth σ-moment and the last two have no simple meaning. For the purpose of this study, the inclusion criteria were fulfilled by third-, fifth-, and sixth-order σ-moments.

The successful calculation of all molecular descriptors with the aid of COSMOtherm [59] requires a proper representation of the molecular structure. This step is performed only once and our database comprises tens of thousands of compounds prepared for use with the BP_TZVPD_FINE_21.ctd parametrization. This step is described in every paper dealing with COSMO-RS computations, so here only a reminder is given that the COSMOconf program (Version 22.0.0) [72] was used for generating the most representative conformers and the geometries were optimized using Turbomole (Version 7.6.0) [73,74].

## 4. Conclusions

This study investigated the solubility of edaravone both experimentally and theoretically. An effort was made to ensure that the solubility data collection was representative and coherent. This is a crucial step for machine learning purposes, aimed at reducing the noise of the data used for model development. The main idea behind the whole project was an extensive exploration of the solubility space by taking edaravone as an exemplary drug.

Finally, it is worth emphasizing that the ensemble of regression models developed in this study was tailored to the physicochemical properties of edaravone solubility by tuning the values of their parameters to a restricted solubility set of this particular drug. While this approach may appear limited to a specific system, it still offers broad generalization potential. In machine learning development, there are generally two philosophies that are not necessarily mutually exclusive. The first one aims for generalization across a broad set of systems but requires a vast amount of experimental data. The second approach restricts itself to a narrower range of systems but is more pragmatic by accepting the scarcity of available measurements. Both approaches share the common tenet of non-linear relationships between the target property and known features. Solubility is one such complex physicochemical property that is dependent on many solute–solvent-related interrelationships. In this study, we offer a balance between these two main attitudes with a pragmatic approach. Our Python code, which utilizes comprehensive parameter tuning, can be used to solve a variety of practical problems encountered in real-life screening. The application of our protocol led to the conclusion that the solubility space defined by ordinary solvents is limited, and it is unlikely to find solvents that are better suited for edaravone dissolution than those depicted in this manuscript. This is not a negative or restrictive conclusion; on the contrary, it points out that this direction is not worth the effort and that focusing on other possibilities might be a better solution. The solubility space is vast and extensive, and one can consider many more potential systems than just common solvents if one is open to accepting new, designed solvents that take advantage of ion pairs. Indeed, such a direction was previously suggested [45], and this study further supports this lineage of future work.

The theoretical framework presented in this study, along with the previous work [28], provides a more precise guide for conducting experiments, saving time and resources in the pursuit of new findings.

## Figures and Tables

**Table 1:** Five top-ranked solvents, selected from the PARIS III collection (EI < 1.0) [28,53], most suited for EDA dissolution. In parentheses, values predicted by the COSMO-RS approach are given. The x_Eest_ symbol denotes the estimated EDA solubility values.

Solvent [CAS number]	Structure	Log (x_Eest_)	EI (PCOP = 0)
enflurane [13838-16-9]	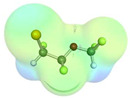	–1.20 ± 0.42 (–1.29)	0.47
DMSO [67-68-5]	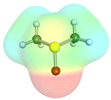	–1.22 ± 0.20 (–1.28)	0.26
isoflurane [26675-46-7]	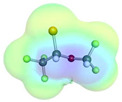	–1.29 ± 0.46 (–1.05)	0.56
NMP [872-50-4]	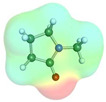	–1.40 ± 0.09 (–0.92)	0.97
2-ethenoxyethanol [764-48-7]	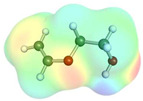	–1.41 ± 0.09 (–1.35)	0.97

## Data Availability

All data supporting the reported results are available on request from the corresponding author.

## Supplementary Materials

The following supporting information can be downloaded at: https://www.mdpi.com/article/10.3390/molecules28196877/s1, (a) in document file: S1. New edaravone solubility data, S2. Solubility data curation, S3. Regressor models’ characteristics; (b) in excel file SM_models.xlsx: molecular descriptors, tuner regressors’ parameters, their predictions, and ensemble definition and prediction.
