# Supplementary material for: Exploring the Solubility Limits of Edaravone in Neat Solvents and Binary Mixtures: Experimental and Machine Learning Study
Source: Molecules. 2023 Sep 29;28(19):6877. doi: 10.3390/molecules28196877 (PMC10574143; doi:10.3390/molecules28196877)
Supplement: Supplementary file 1 [file molecules-28-06877-s001.zip › Supplementary Materials.pdf]

**<u>Supplementary Materials</u>**

# Exploring the solubility limits of Edaravone in neat solvents and binary mixtures: experimental and machine learning study

Maciej Przybyłek, Tomasz Jeliński, Magdalena Mianowana, Kinga Misiak, Piotr Cysewski
Department of Physical Chemistry, Pharmacy Faculty, Collegium Medicum of Bydgoszcz, Nicolaus Copernicus University in Toruń, Kurpińskiego 5, 85-096 Bydgoszcz, Poland

## Table of Contents

S1. New Edaravone solubility data ........ 2

Table S1. The experimentally determined mole fraction solubility of Edaravone ($x_{EDA}$, $\times10^2$) in diglyme (DIG), triglyme (TIG), tetraglyme (TEG), dimethyl sulfoxide (DMSO), 1-methyl-2-pyrrolidone, (NMP), 4-formylmorpholine (4FM), and 2,4-dimethylphenol (DMP) at various temperatures. Standard deviation values are given in parentheses. ........ 2

Table S2. The experimentally determined mole fraction solubility of Edaravone ($x_{EDA}$, $\times10^2$) in 1,2-propanediol (PG), diethylene glycol (DG), triethylene glycol (TG), and 1,3-butanediol (BG), as well as in their aqueous binary mixtures at 25 °C. In the first column, $x_2$* denotes the mole fraction of the organic solvent in solute-free solutions. Standard deviation values are given in parentheses. ........ 2

Figure S1. The DSC (a) and FTIR-ATR (b) characteristics of the solid residues obtained after shake-flask solubility determination procedure. Notation is the same as in Tables S1. and S2. .... 3

S2. Solubility data curation ........ 4

S2.1. Collection of EDA solubility in neat solvents ........ 4

Table S3. Optimized values of model parameters used for data curation along with statistical measure of the fitting accuracy. ........ 4

Figure S2. Graphical illustration of the solubility data curation of edaravone in neat solvents. The black diamonds represent curated values constituting the solubility dataset, while gray symbols characterize experimental points [a], [b], [c] taken from [Li et al., J. Chem. Thermodyn. 2019, 138, 304–312], [Qiu et al., J. Chem. Eng. Data 2020, 65, 3240–3251], [Cysewski et al., Molecules 2023, 28, 629], respectively. ........ 7

S2.2. Binary solvents mixtures ........ 7

Table S4. Solubility data curation of edaravone in binary solvents. All experimental data come from reference 18 as cited in main text or from this work. The $x_E$ and $x_2$* symbols denote mole fraction solubility of EDA and mole fraction of component 2 in solute-free solutions, respectively. ........ 7

S3. Regressor models characteristics ........ 15

Table S5. Graphical illustration of the of the regression model performance belonging to set A. The panels a,b, and c document the correlation between computed and consensus solubility values with annotation of the standard deviation as circles radius, applicability domain plots, and results of learning curve analysis concerning both $R^2$ and MAE, respectively. ........ 15

Table S6. Graphical illustration of the of the regression model performance belonging to set B. The panels a,b, and c document the correlation between computed and consensus solubility values with annotation of the standard deviation as circles radius, applicability domain plots, and results of learning curve analysis concerning both $R^2$ and MAE, respectively. ........ 18

## S1. New Edaravone solubility data

**Table S1. The experimentally determined mole fraction solubility of Edaravone ($x_{EDA}$, ×10$^2$) in diglyme (DIG), triglyme (TIG), tetraglyme (TEG), dimethyl sulfoxide (DMSO), 1-methyl-2-pyrrolidone, (NMP), 4-formylmorpholine (4FM), and 2,4-dimethylphenol (DMP) at various temperatures. Standard deviation values are given in parentheses.**

| solvent | Edaravone solubility ($x_{EDA}$, × 10$^2$) | | | |
|---|---|---|---|---|
| | 25 °C | 30 °C | 35 °C | 40 °C |
| DMSO | 7.58 (±0.32) | 12.25 (±0.16) | 20.1 (±0.15) | 29.81 (±1.14) |
| TEG | 4.63 (±0.05) | 8.63 (±0.22) | 13.05 (±0.19) | 21.21 (±0.64) |
| NMP | 3.83 (±0.01) | 4.96 (±0.49) | 6.94 (±0.19) | 8.61 (±0.21) |
| DIG | 2.05 (±0.02) | 2.69 (±0.04) | 3.33 (±0.11) | 4.16 (±0.06) |
| DMP | 1.88 (±0.05) | 2.82 (±0.58) | 4.08 (±0.21) | 5.65 (±0.25) |
| TIG | 1.53 (±0.07) | 2.20 (±0.04) | 3.11 (±0.17) | 4.57 (±0.39) |
| 4FM | 1.11 (±0.01) | 1.46 (±0.08) | 1.76 (±0.19) | 2.17 (±0.01) |

**Table S2. The experimentally determined mole fraction solubility of Edaravone ($x_{EDA}$, ×10$^2$) in 1,2-propanediol (PG), diethylene glycol (DG), triethylene glycol (TG), and 1,3-butanediol (BG), as well as in their aqueous binary mixtures at 25 °C. In the first column, $x_2$* denotes the mole fraction of the organic solvent in solute-free solutions. Standard deviation values are given in parentheses.**

| $x_2$* | Edaravone solubility ($x_{EDA}$, × 10$^2$) | | | |
|---|---|---|---|---|
| | PG | DG | TG | BG |
| 0.0 | 0.02 (<±0.01) | 0.02 (<±0.01) | 0.02 (<±0.01) | 0.02 (<±0.01) |
| 0.1 | 0.06 (±0.01) | 0.09 (±0.01) | 0.31 (±0.04) | 0.12 (±0.02) |
| 0.2 | 0.15 (±0.01) | 0.25 (±0.01) | 0.78 (±0.03) | 0.29 (±0.04) |
| 0.3 | 0.26 (±0.02) | 0.48 (±0.04) | 1.28 (±0.07) | 0.53 (±0.03) |
| 0.4 | 0.40 (±0.02) | 0.74 (±0.03) | 1.72 (±0.06) | 0.83 (±0.04) |
| 0.5 | 0.54 (±0.02) | 1.09 (±0.01) | 2.11 (±0.08) | 1.07 (±0.05) |
| 0.6 | 0.71 (±0.02) | 1.39 (±0.02) | 2.54 (±0.08) | 1.27 (±0.03) |
| 0.7 | 0.86 (±0.02) | 1.65 (±0.05) | 3.03 (±0.09) | 1.42 (±0.05) |
| 0.8 | 0.99 (±0.02) | 1.87 (±0.04) | 3.51 (±0.09) | 1.50 (±0.03) |
| 0.9 | 1.00 (±0.02) | 1.98 (±0.02) | 3.58 (±0.06) | 1.51 (±0.04) |
| 1.0 | 0.85 (±0.01) | 2.04 (±0.06) | 2.75 (±0.08) | 1.43 (±0.04) |

a

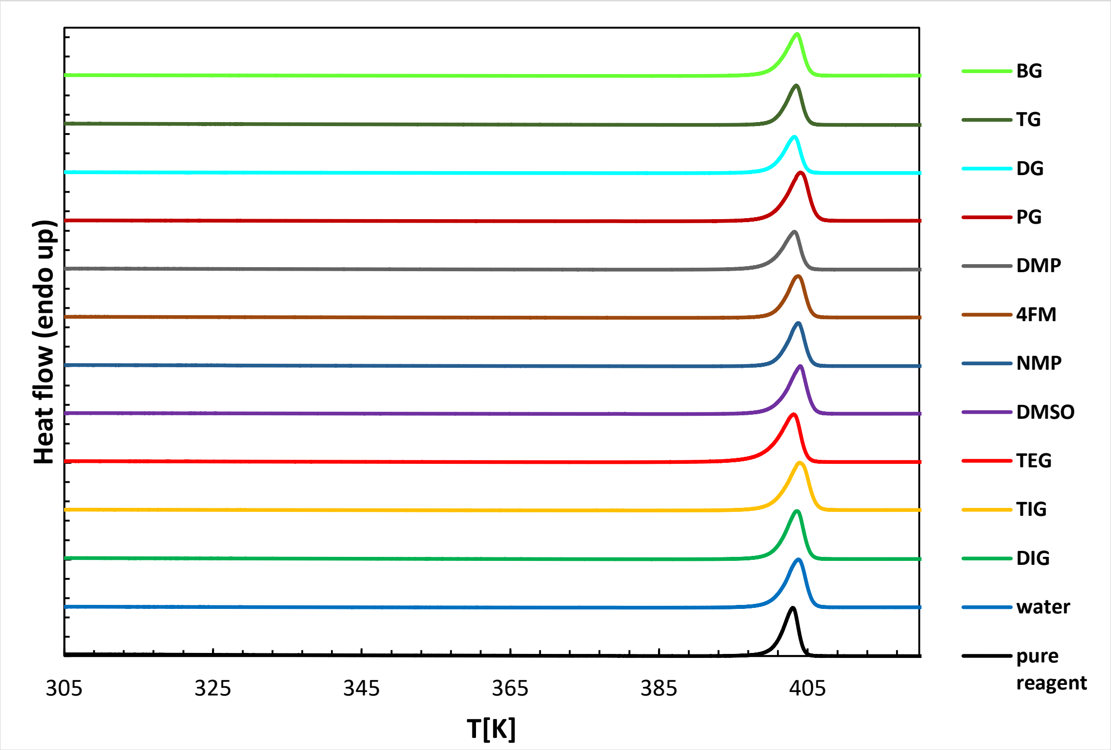


b

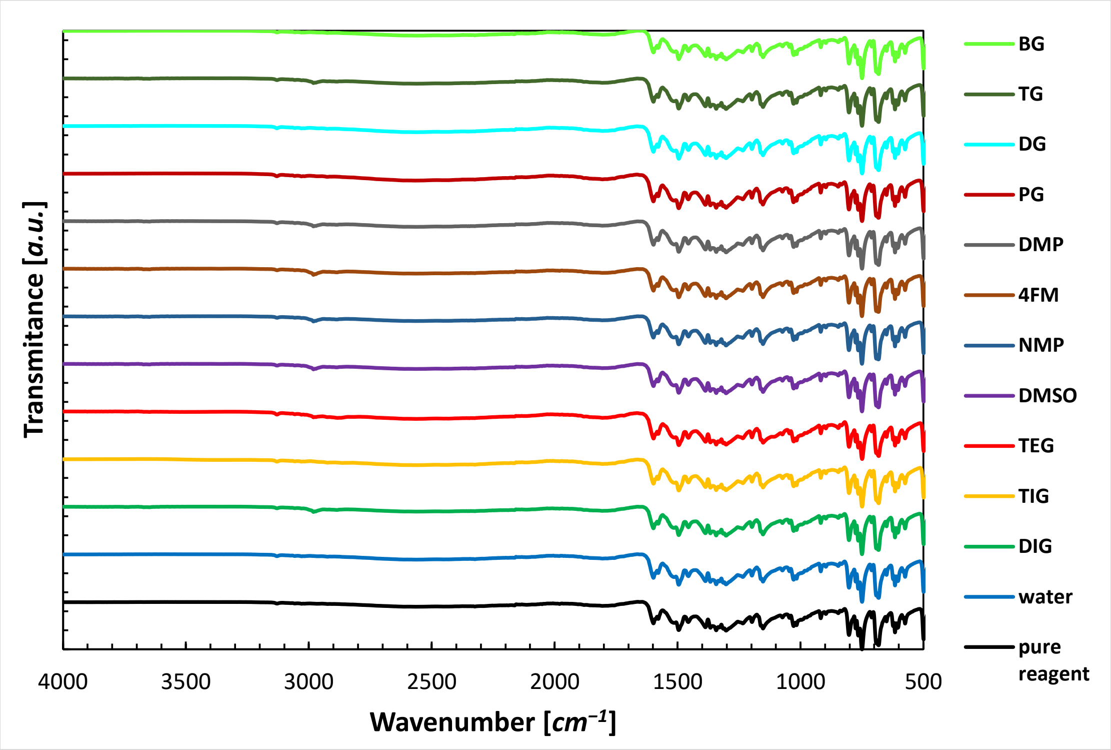


**Figure S1. The DSC (a) and FTIR-ATR (b) characteristics of the solid residues obtained after shake-flask solubility determination procedure. Notation is the same as in Tables S1. and S2.**

## S2. Solubility data curation

### *S2.1. Collection of EDA solubility in neat solvents*

**Table S3. Optimized values of model parameters used for data curation along with statistical measure of the fitting accuracy.**

| solvent | acronim | A × $10^3$ | B × $10^3$ | C × $10^3$ | RMSD × $10^3$ | MAPE |
|---|---|---|---|---|---|---|
| water | W | 3.111 | -6.398 | 0.858 | 4.03 | 0.04 |
| methanol | MeOH | 31.984 | -17.949 | 2.185 | 165.47 | 4.19 |
| ethanol | EtOH | 20.389 | -11.949 | 1.439 | 68.09 | 1.33 |
| n-propanol | nPrOH | 29.274 | -17.128 | 2.198 | 133.95 | 2.51 |
| isopropanol | iPrOH | 18.599 | -10.825 | 1.246 | 90.26 | 1.90 |
| n-butanol | nBuOH | 15.486 | -8.444 | 0.834 | 8.89 | 0.24 |
| isobutanol | iBuOH | 21.045 | -11.898 | 1.348 | 18.43 | 0.45 |
| sec-butanol | sBuOH | 20.873 | -11.790 | 1.332 | 11.53 | 0.30 |
| n-pentanol | nPeOH | 34.206 | -20.257 | 2.695 | 14.61 | 0.34 |
| acetone | AT | 49.838 | -28.045 | 3.600 | 192.27 | 4.65 |
| acetonitrile | AE | 8.189 | -3.658 | 0.000 | 58.89 | 1.22 |
| dichloromethane | DC | 40.161 | -22.691 | 2.956 | 11.54 | 0.36 |
| ethyl acetate | EA | 44.601 | -26.411 | 3.577 | 178.67 | 4.16 |
| 1,4-dioxane | D | 17.497 | -9.079 | 0.865 | 21.92 | 0.68 |
| toluene | T | 3.251 | -1.145 | -0.296 | 7.08 | 0.16 |
| 2,4-dimethylphenol | DMP | -16.508 | 14.856 | -3.315 | 3.76 | 0.10 |
| 1-methyl-2-pyrrolidone | NMP | 10.517 | -3.004 | -0.330 | 23.14 | 0.73 |
| diglyme | DIG | -12.269 | 9.697 | -2.146 | 7.41 | 0.18 |
| triglyme | TIG | 62.116 | -33.404 | 4.066 | 5.93 | 0.14 |
| tetraglyme | TEG | -31.442 | 27.130 | -5.565 | 32.42 | 1.16 |
| DMSO | DMSO | -9.106 | 13.021 | -3.302 | 14.09 | 0.74 |
| 4FM | 4FM | 9.308 | -4.112 | 0.000 | 15.91 | 0.31 |

**Water (W)**

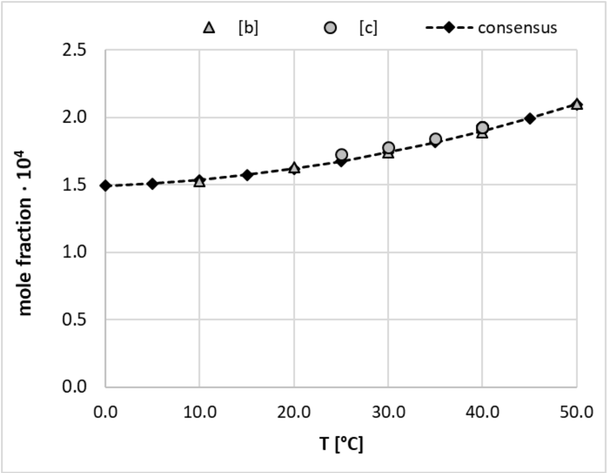


**Methanol (MeOH)**

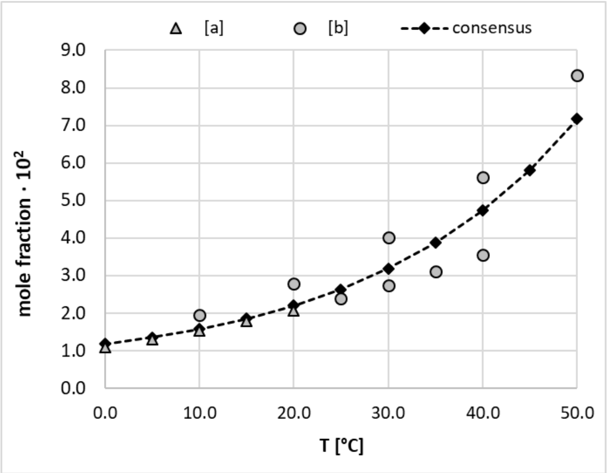


**Ethanol (EtOH)**

**n-Propanol (nPrOH)**

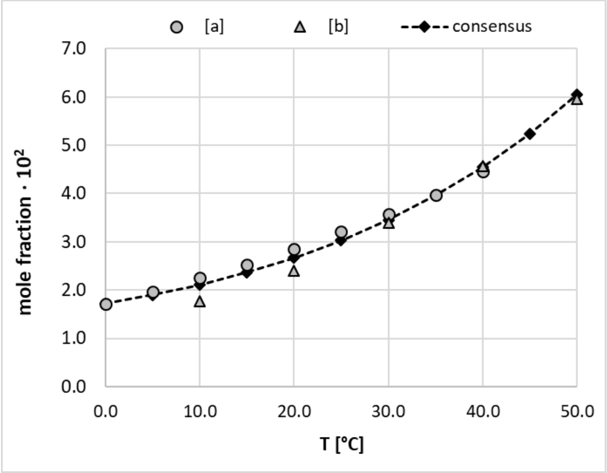


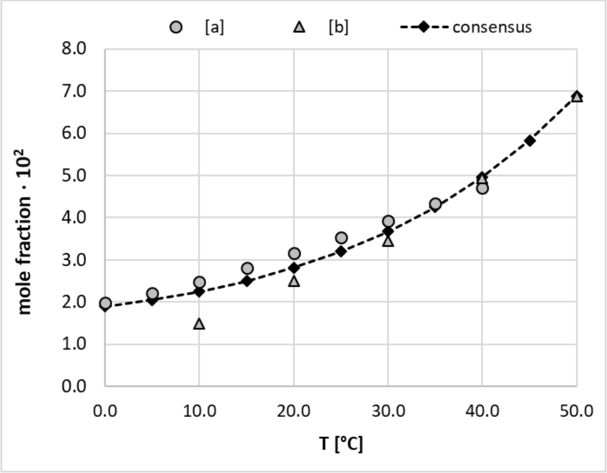


**Isopropanol (iPrOH)**

**n-Butanol (nBuOH)**

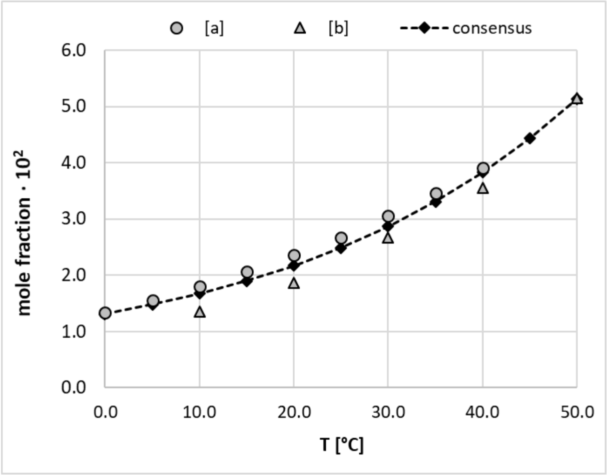


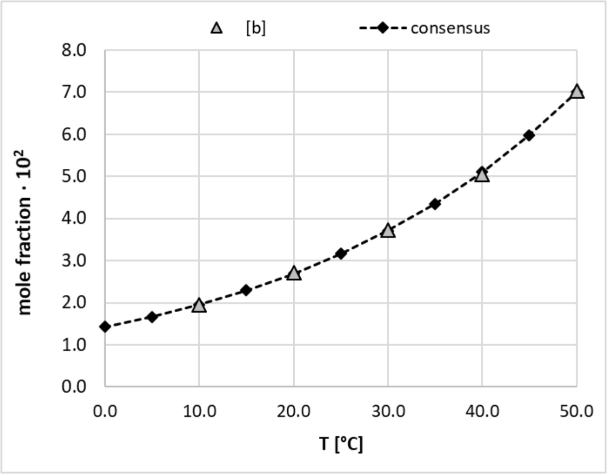


**Isobutanol (iBuOH)**

**sec-Butanol (sBuOH)**

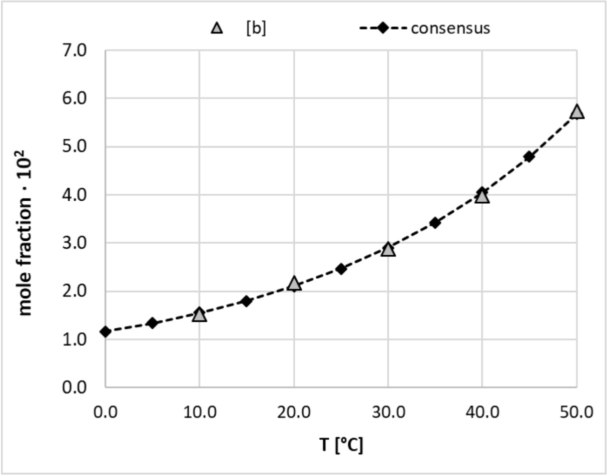


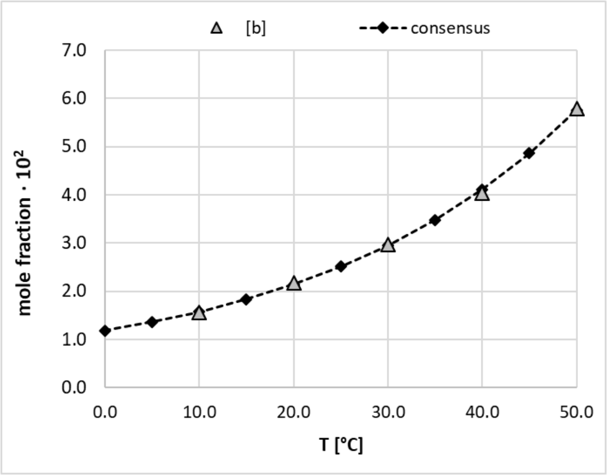


**n-Pentanol (nPeOH)**

**Acetone (Ac)**

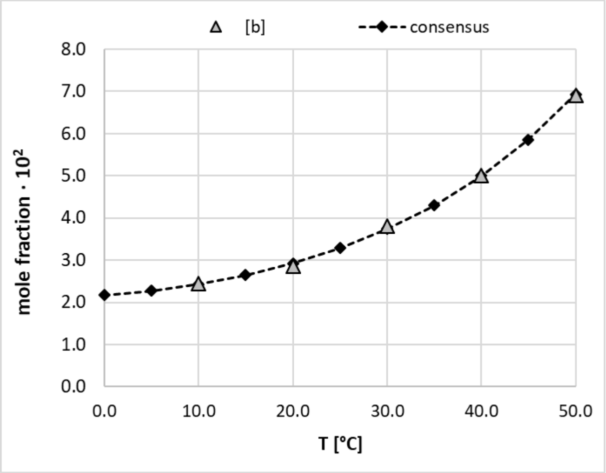


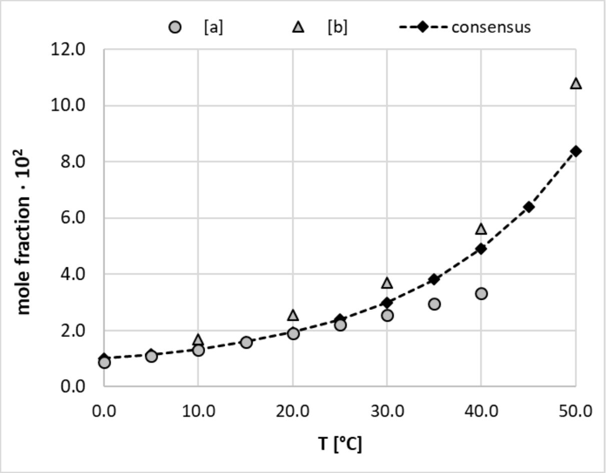


**Acetonitrile** | **Dichloromethane**

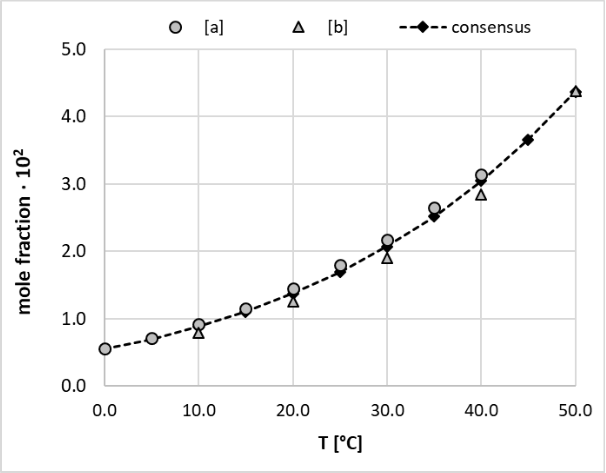


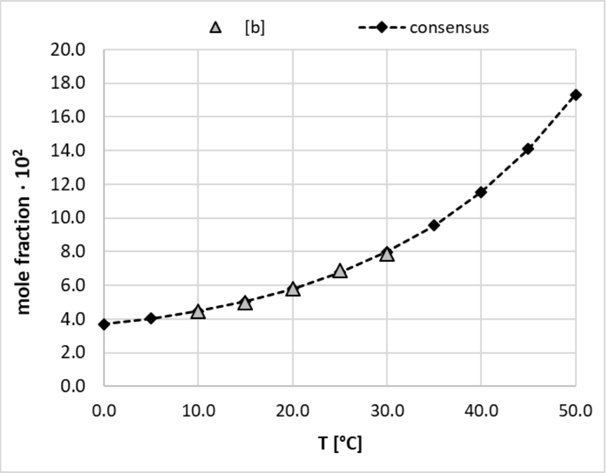


**Ethyl Acetate(EtAc)** | **1,4-Dioxane (D)**

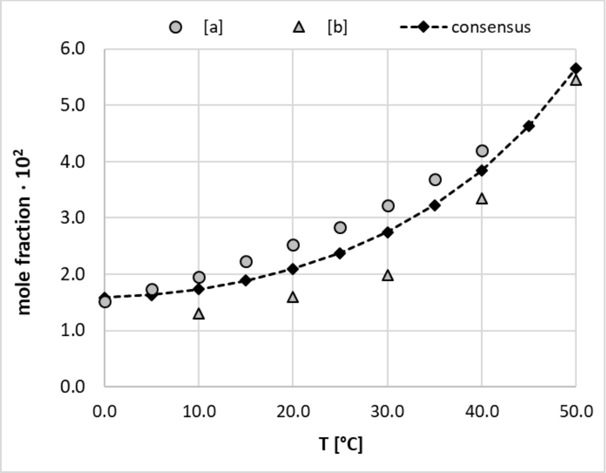


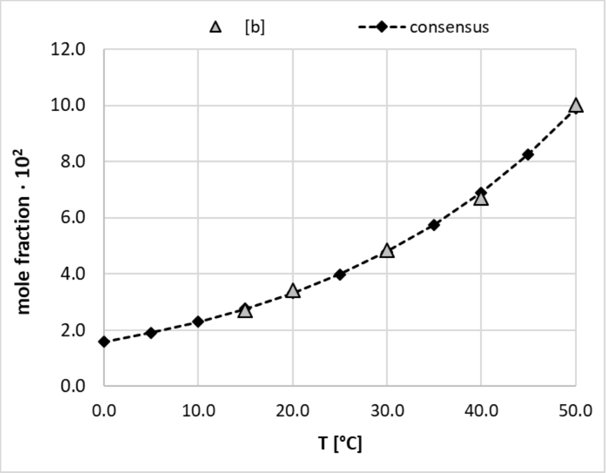


**Toluene (T)**

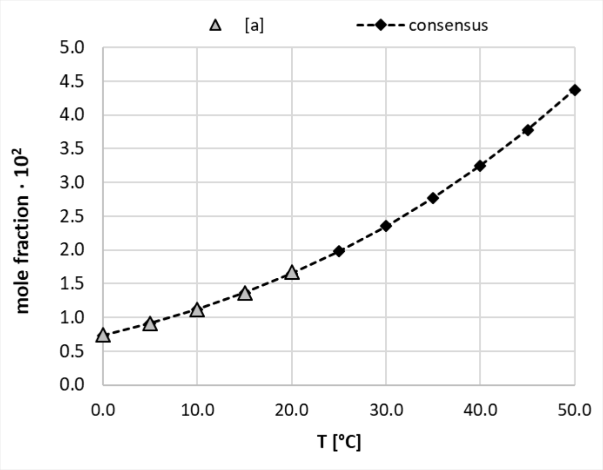


**Figure S2. Graphical illustration of the solubility data curation of edaravone in neat solvents. The black diamonds represent curated values constituting the solubility dataset, while gray symbols characterize experimental points [a], [b], [c] taken from [Li et al., J. Chem. Thermodyn. 2019, 138, 304–312], [Qiu et al., J. Chem. Eng. Data 2020, 65, 3240–3251], [Cysewski et al., Molecules 2023, 28, 629], respectively.**

### *S2.2. Binary solvents mixtures*

$$\ln x_{\mathrm{w,T}} = w_1 \ln x_{1,\mathrm{T}} + w_2 \ln x_{2,\mathrm{T}} + \frac{w_1 w_2}{T/\mathrm{K}} \sum_{\mathrm{i}=0}^{2} J_{\mathrm{i}} (w_1 - w_2)^{\mathrm{i}}$$

**Table S4. Solubility data curation of edaravone in binary solvents. All experimental data come from reference 18 as cited in main text or from this work. The $x_E$ and $x_2$* symbols denote mole fraction solubility of EDA and mole fraction of component 2 in solute-free solutions, respectively.**

**water (2) – methanol (3) [Li et al., J. Chem. Thermodyn. 2019, 138, 304–312]**

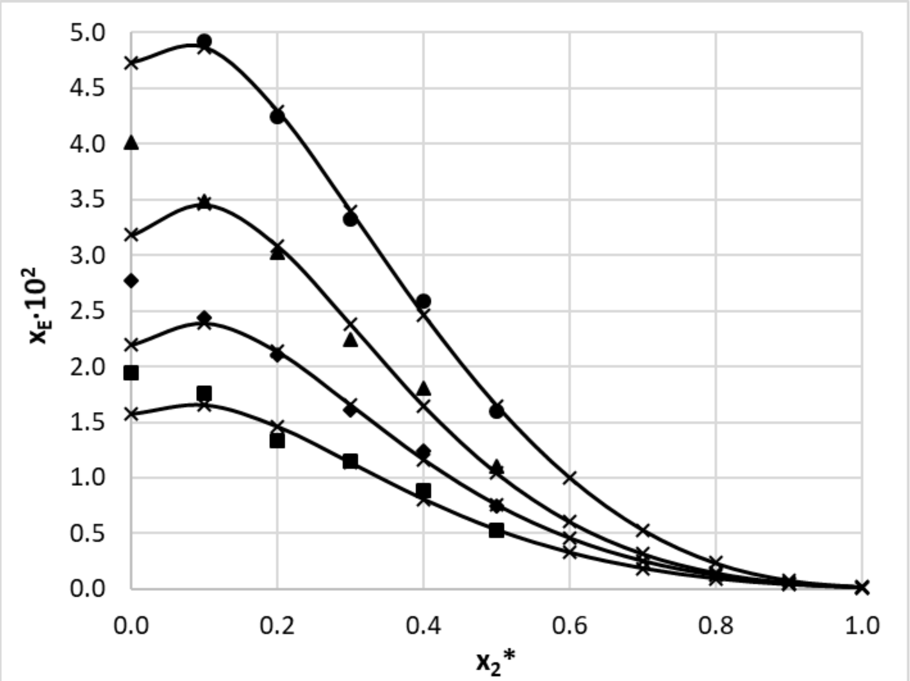


| *T*[°C] | $J_0$ | $J_1$ | $J_2$ | RMSD | MAPE |
|---|---|---|---|---|---|
| 10.0 | 4.935 | 0.320 | 1.592 | 58.959 | 14.259 |
| 20.0 | 5.576 | 0.562 | 1.995 | 36.531 | 8.931 |

| | | | | | |
|---|---|---|---|---|---|
| 30.0 | 5.955 | 0.417 | 1.703 | 72.284 | 4.090 |
| 40.0 | 6.824 | 2.131 | 2.068 | 52.056 | 3.162 |
| 50.0 | 7.632 | 2.241 | 1.469 | 119.003 | 4.342 |

**water (2)- ethanol (3) [Li et al., J. Chem. Thermodyn. 2019, 138, 304–312]**

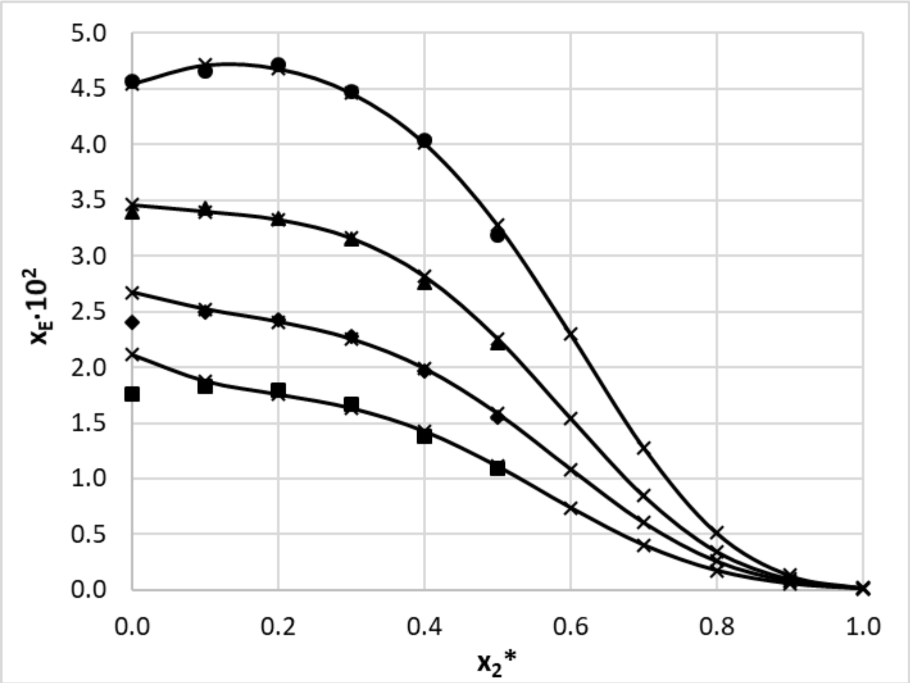


| *T*[°C] | *$J_0$* | *$J_1$* | *$J_2$* | RMSD | MAPE |
|---|---|---|---|---|---|
| 10.0 | 7.281 | 3.449 | -0.574 | 32.544 | 6.264 |
| 20.0 | 8.122 | 4.338 | 0.587 | 30.913 | 5.251 |
| 30.0 | 8.877 | 4.757 | 0.964 | 49.154 | 5.418 |
| 40.0 | 9.646 | 5.615 | 2.082 | 58.181 | 5.770 |
| 50.0 | 10.520 | 6.562 | 3.602 | 87.403 | 6.526 |

**n-propanol (3) –acetonitrile (2) [Li et al., J. Chem. Thermodyn. 2019, 138, 304–312]**

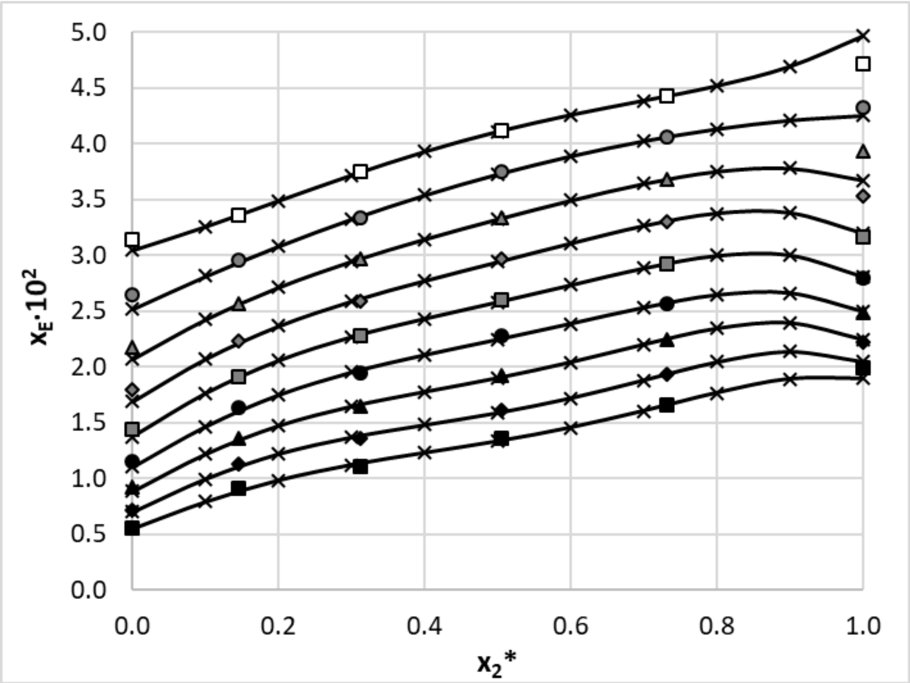


| *T*[°C] | *$J_0$* | *$J_1$* | *$J_2$* | RMSD | MAPE |
|---|---|---|---|---|---|

| | | | | | |
|---|---|---|---|---|---|
| 0.0 | 1.069 | -0.816 | 1.467 | 15.902 | 1.065 |
| 5.0 | 1.131 | -0.666 | 1.658 | 12.582 | 0.691 |
| 10.0 | 1.198 | -0.494 | 1.470 | 13.761 | 0.629 |
| 15.0 | 1.204 | -0.398 | 1.128 | 19.602 | 0.756 |
| 20.0 | 1.092 | -0.275 | 1.030 | 6.988 | 0.228 |
| 25.0 | 0.936 | -0.138 | 0.778 | 13.604 | 0.392 |
| 30.0 | 0.746 | -0.106 | 0.453 | 3.783 | 0.096 |
| 35.0 | 0.521 | -0.109 | 0.060 | 13.166 | 0.294 |
| 40.0 | 0.223 | -0.180 | -0.249 | 1.361 | 0.027 |

**n-propanol (3) -ethyl acetate (2) [Li et al., J. Chem. Thermodyn. 2019, 138, 304–312]**

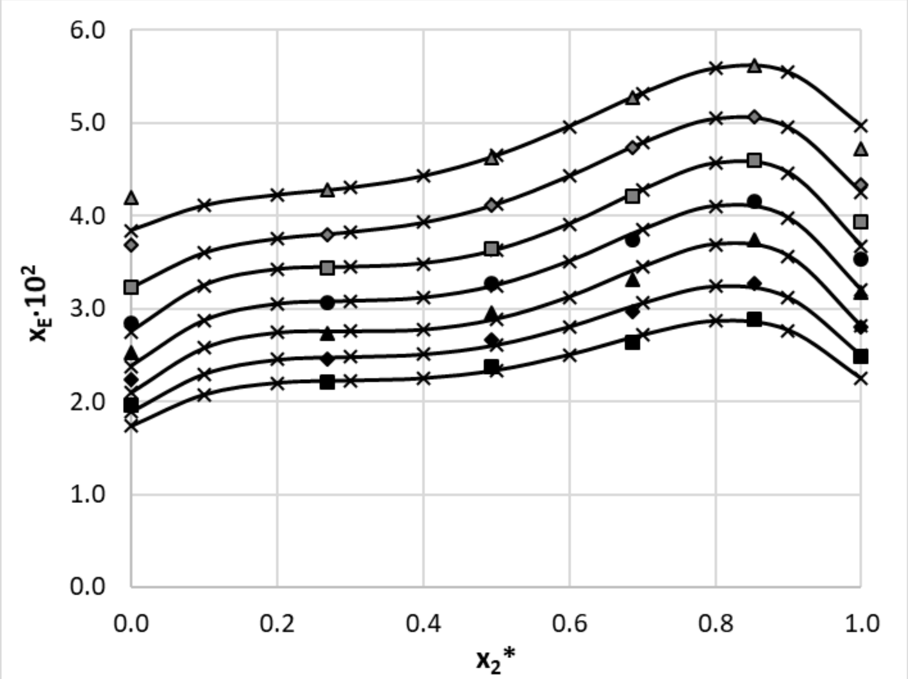


| *T*[°C] | *$J_0$* | *$J_1$* | *$J_2$* | **RMSD** | **MAPE** |
|---|---|---|---|---|---|
| 0.0 | 0.348 | 0.736 | 0.694 | 2.541 | 0.094 |
| 5.0 | 0.523 | 0.591 | 1.736 | 17.786 | 0.608 |
| 10.0 | 0.674 | 0.563 | 2.305 | 32.779 | 0.989 |
| 15.0 | 0.737 | 0.579 | 2.493 | 41.052 | 1.104 |
| 20.0 | 0.693 | 0.629 | 2.765 | 54.727 | 1.314 |
| 25.0 | 0.655 | 0.618 | 2.495 | 34.850 | 0.716 |
| 30.0 | 0.531 | 0.592 | 2.309 | 15.065 | 0.290 |
| 35.0 | 0.427 | 0.671 | 1.604 | 5.045 | 0.087 |
| 40.0 | 0.246 | 0.652 | 1.153 | 2.607 | 0.040 |

**ethanol (3) –acetonitrile (2) [Li et al., J. Chem. Thermodyn. 2019, 138, 304–312]**

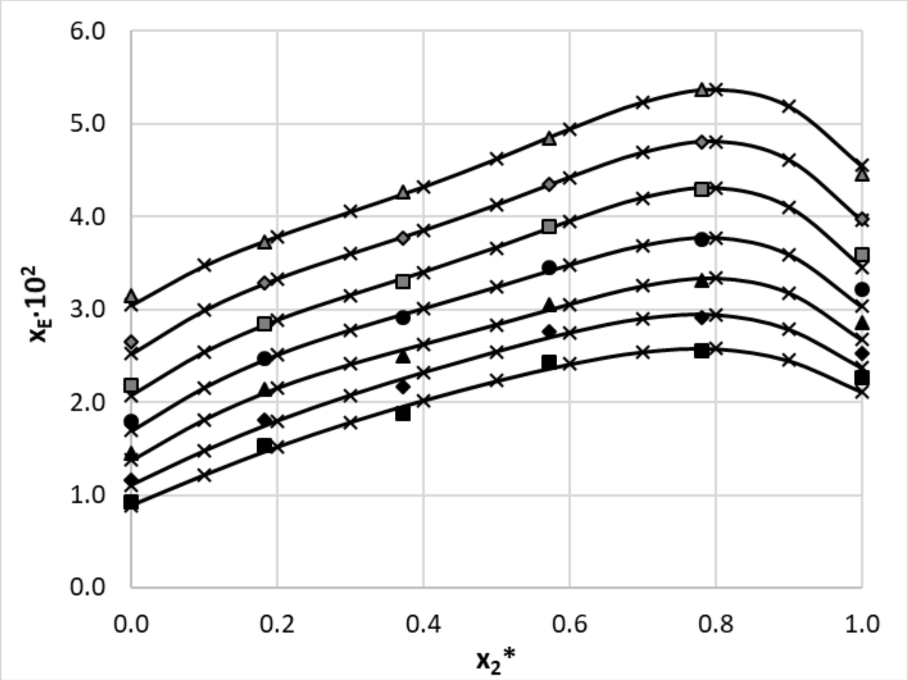


| *T*[°C] | *J₀* | *J₁* | *J₂* | RMSD | MAPE |
|---|---|---|---|---|---|
| 0.0 | 2.244 | -0.142 | 0.369 | 16.928 | 0.823 |
| 5.0 | 2.130 | -0.042 | 0.741 | 27.080 | 1.264 |
| 10.0 | 1.959 | 0.039 | 0.986 | 49.030 | 1.951 |
| 15.0 | 1.810 | 0.182 | 1.075 | 50.854 | 1.740 |
| 20.0 | 1.570 | 0.198 | 1.470 | 39.716 | 1.175 |
| 25.0 | 1.433 | 0.297 | 1.346 | 25.193 | 0.621 |
| 30.0 | 1.255 | 0.491 | 1.274 | 18.085 | 0.407 |
| 35.0 | 1.066 | 0.501 | 1.135 | 4.698 | 0.093 |
| 40.0 | 0.865 | 0.566 | 0.927 | 10.274 | 0.180 |

**ethanol (3) -ethyl acetate (2) [Li et al., J. Chem. Thermodyn. 2019, 138, 304–312]**

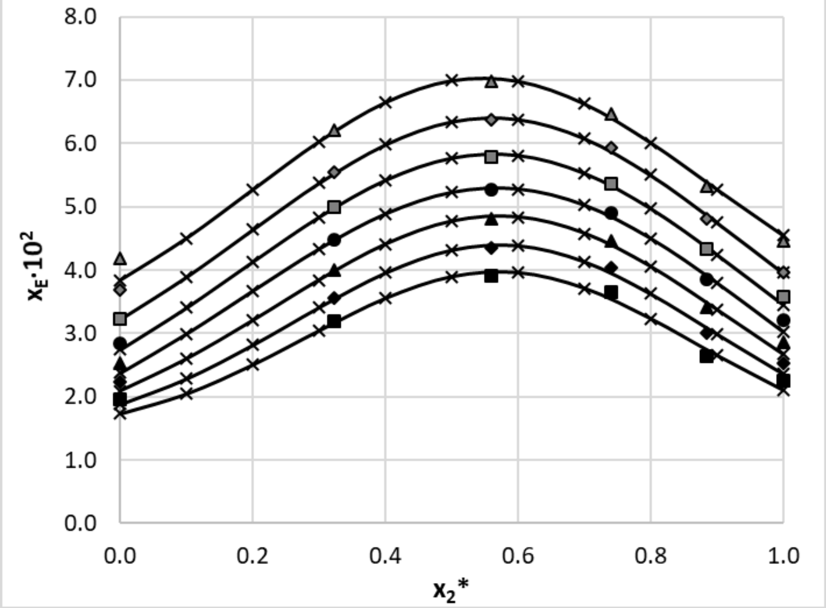


| *T*[°C] | *J₀* | *J₁* | *J₂* | RMSD | MAPE |
|---|---|---|---|---|---|
| 0.0 | 2.559 | 0.951 | -1.637 | 102.143 | 2.974 |

| | | | | | |
|---|---|---|---|---|---|
| 5.0 | 2.792 | 0.817 | -1.586 | 91.718 | 2.223 |
| 10.0 | 2.843 | 0.714 | -0.996 | 68.643 | 1.564 |
| 15.0 | 2.860 | 0.601 | -0.737 | 55.115 | 1.117 |
| 20.0 | 2.805 | 0.463 | -0.471 | 47.899 | 0.867 |
| 25.0 | 2.665 | 0.301 | -0.192 | 35.629 | 0.579 |
| 30.0 | 2.503 | 0.246 | -0.261 | 27.505 | 0.393 |
| 35.0 | 2.296 | 0.247 | -0.361 | 37.908 | 0.502 |
| 40.0 | 2.058 | 0.159 | -0.546 | 47.431 | 0.570 |

**methanol (3) -acetonitrile(2) [Li et al., J. Chem. Thermodyn. 2019, 138, 304–312]**

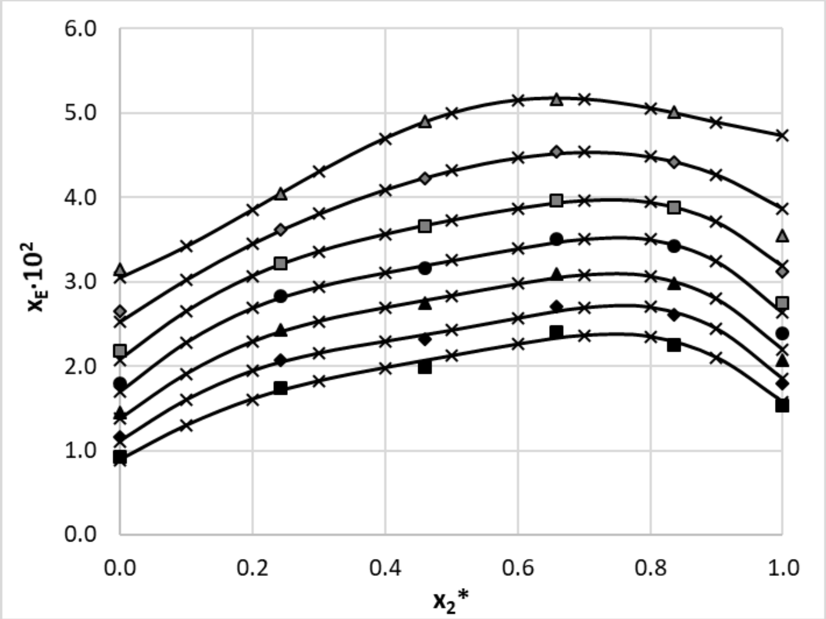


| *T*[°C] | *J₀* | *J₁* | *J₂* | RMSD | MAPE |
|---|---|---|---|---|---|
| 0.0 | 2.752 | -0.076 | 1.068 | 24.958 | 1.287 |
| 5.0 | 2.534 | 0.047 | 1.954 | 40.614 | 1.634 |
| 10.0 | 2.350 | 0.179 | 2.151 | 49.757 | 1.839 |
| 15.0 | 2.113 | 0.109 | 2.314 | 36.525 | 1.162 |
| 20.0 | 1.960 | 0.051 | 1.891 | 26.510 | 0.726 |
| 25.0 | 1.726 | -0.005 | 1.693 | 21.156 | 0.504 |
| 30.0 | 1.493 | -0.034 | 1.118 | 12.033 | 0.242 |
| 35.0 | 1.293 | 0.030 | 0.405 | 9.733 | 0.178 |
| 40.0 | 1.098 | 0.043 | -0.434 | 6.343 | 0.102 |

| methanol (3) -ethyl acetate (2) [Li et al., J. Chem. Thermodyn. 2019, 138, 304–312] | | | | | |
|---|---|---|---|---|---|

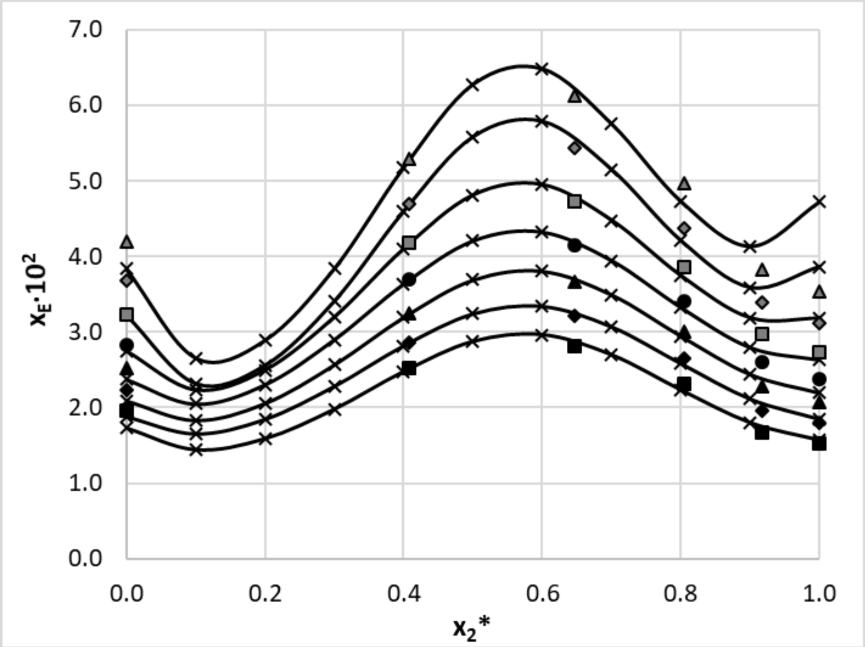


| *T*[°C] | $J_0$ | $J_1$ | $J_2$ | RMSD | MAPE |
|---|---|---|---|---|---|
| 0.0 | 1.765 | 1.825 | -2.584 | 61.288 | 2.725 |
| 5.0 | 2.022 | 1.807 | -2.824 | 52.796 | 2.027 |
| 10.0 | 2.220 | 2.071 | -3.857 | 55.394 | 1.836 |
| 15.0 | 2.209 | 1.805 | -3.397 | 55.023 | 1.589 |
| 20.0 | 2.172 | 1.724 | -3.630 | 57.723 | 1.448 |
| 25.0 | 2.079 | 1.590 | -4.015 | 71.032 | 1.563 |
| 30.0 | 1.948 | 1.661 | -4.848 | 92.465 | 1.790 |
| 35.0 | 1.832 | 2.029 | -6.378 | 117.226 | 2.005 |
| 40.0 | 1.551 | 1.919 | -6.782 | 176.555 | 2.651 |

**water (2) –Isopropanol (3) [Li et al., J. Chem. Thermodyn. 2019, 138, 304–312]**

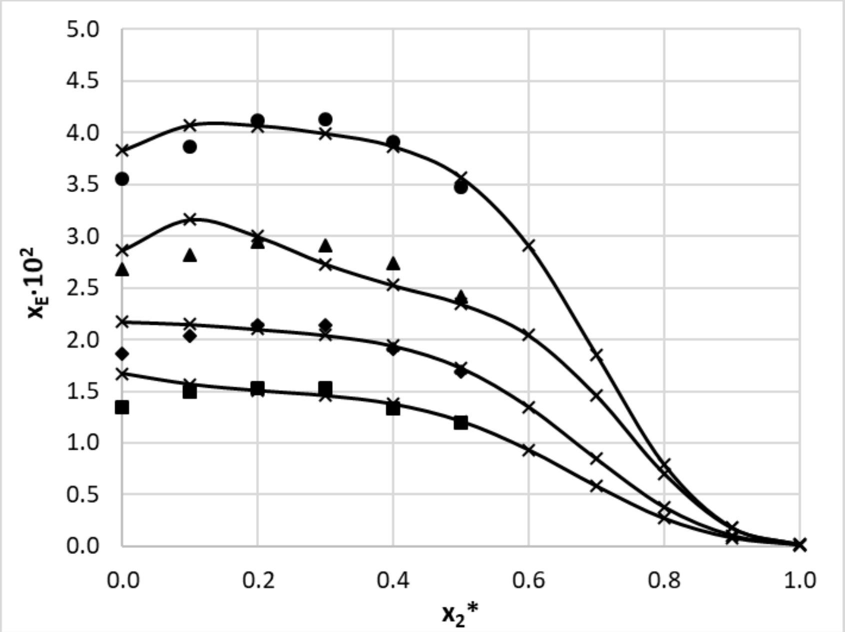


| *T*[°C] | $J_0$ | $J_1$ | $J_2$ | RMSD | MAPE |
|---|---|---|---|---|---|
| 10.0 | 8.087 | 5.722 | 1.542 | 42.478 | 4.391 |

| | | | | | |
|---|---|---|---|---|---|
| 20.0 | 8.881 | 6.437 | 2.481 | 63.409 | 6.063 |
| 30.0 | 9.406 | 8.426 | 6.408 | 149.800 | 5.715 |
| 40.0 | 10.331 | 8.081 | 4.253 | 137.453 | 8.299 |
| 50.0 | 11.214 | 8.878 | 5.335 | 218.016 | 9.134 |

**diethylene glycol (2) -water(3) [this work]**

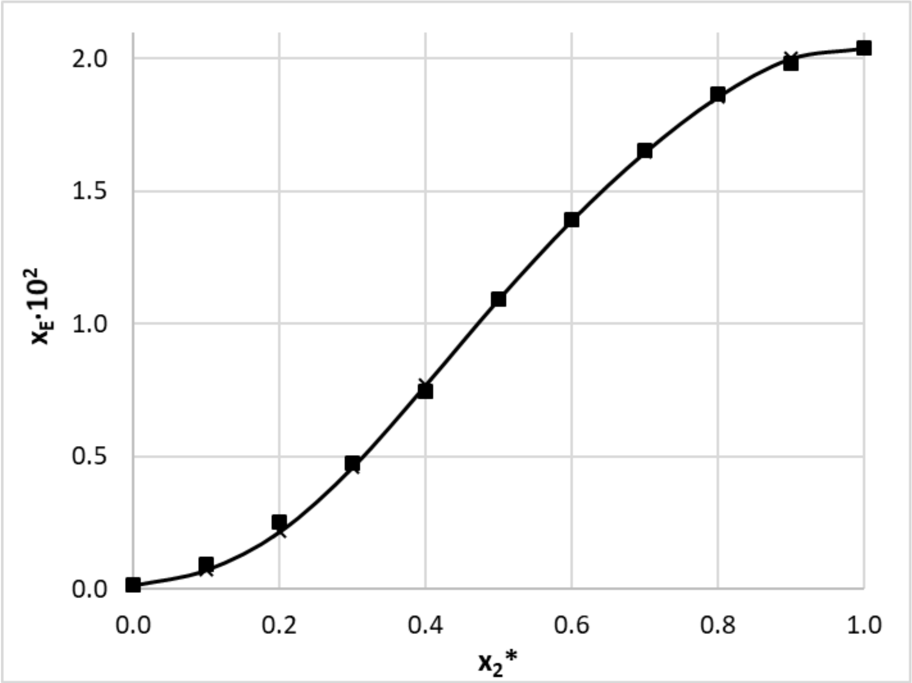


| *T*[°C] | $J_0$ | $J_1$ | $J_2$ | RMSD | MAPE |
|---|---|---|---|---|---|
| 25.0 | 7.112 | -3.865 | 1.720 | 16.725 | 3.815 |

**triethylene glycol (2) –water (3) [this work]**

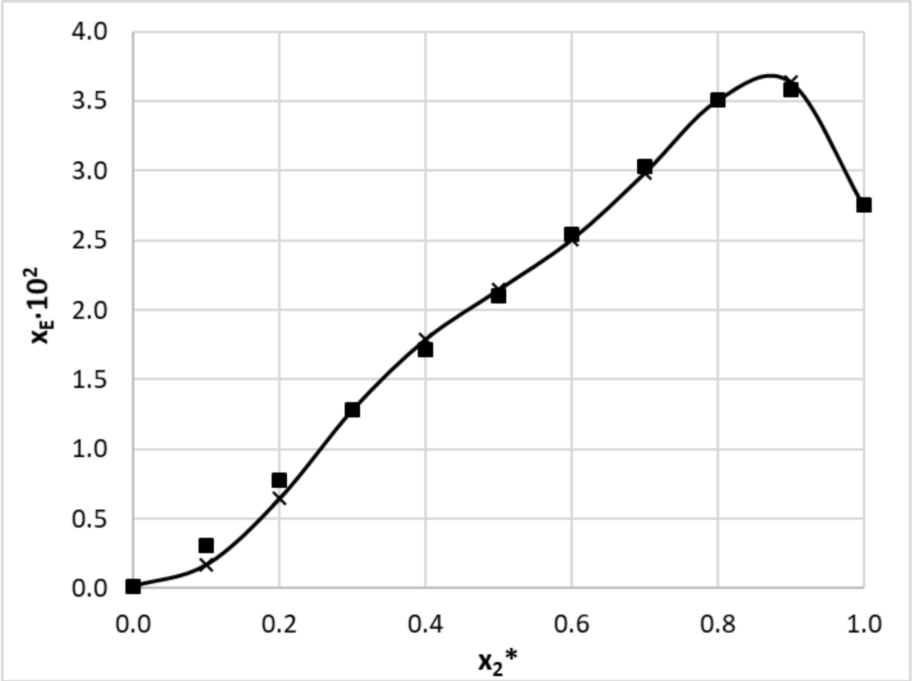


| *T*[°C] | $J_0$ | $J_1$ | $J_2$ | RMSD | MAPE |
|---|---|---|---|---|---|
| 25.0 | 9.207 | -7.124 | 8.221 | 67.058 | 6.571 |

**1,3-butanediol-water-water [this work]**

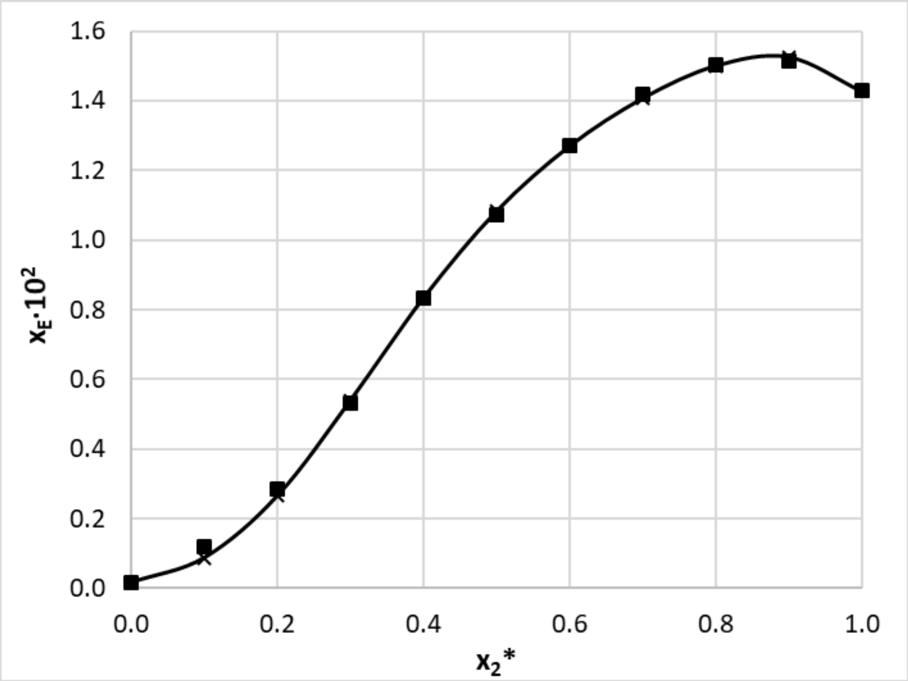


| *T*[°C] | *J₀* | *J₁* | *J₂* | RMSD | MAPE |
|---|---|---|---|---|---|
| 25.0 | 7.787 | -4.889 | 2.818 | 13.222 | 3.480 |

**1,2-propanediol-water [this work]**

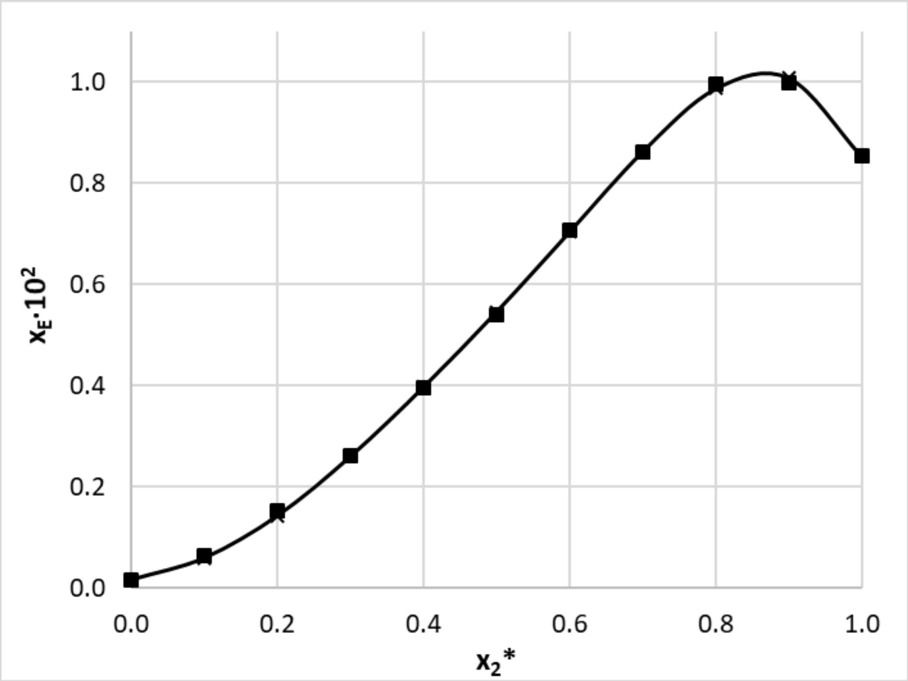


| *T*[°C] | $J_0$ | $J_1$ | $J_2$ | RMSD | MAPE |
|---|---|---|---|---|---|
| 25.0 | 6.077 | -2.239 | 3.021 | 5.422 | 1.480 |

# S3. Regressor models characteristics

***Table S5. Graphical illustration of the of the regression model performance belonging to set A. The panels a,b, and c document the correlation between computed and consensus solubility values with annotation of the standard deviation as circles radius, applicability domain plots, and results of learning curve analysis concerning both $R^2$ and MAE, respectively.***

<table>
<tr><th colspan="2">NuSVR</th></tr>
<tr><th>a</th><th>b</th></tr>
<tr><td>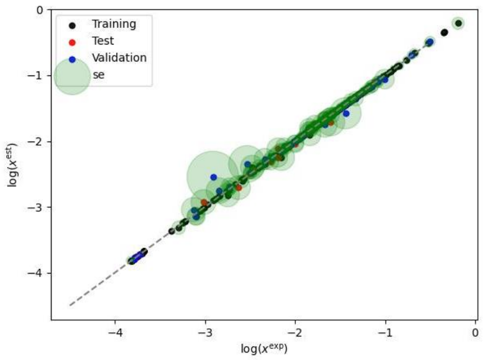
</td><td>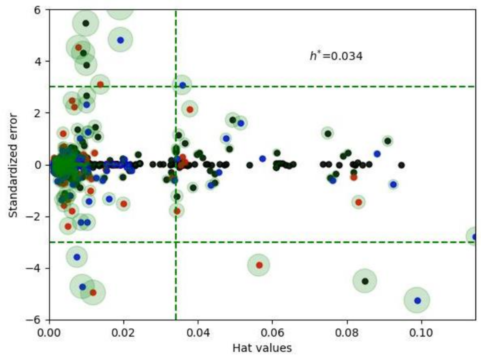
</td></tr>
<tr><th colspan="2">c</th></tr>
<tr><td>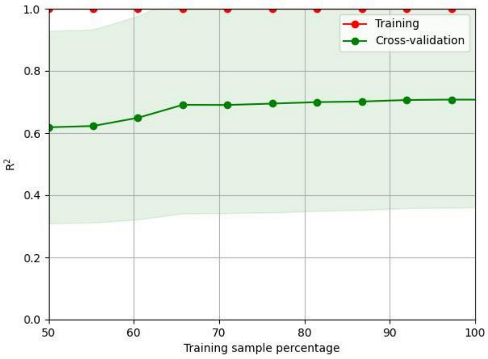
</td><td>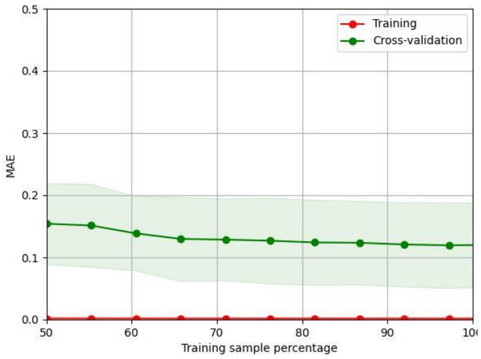
</td></tr>
</table>

<table>
<tr><th colspan="2">SVR</th></tr>
<tr><th>a</th><th>b</th></tr>
<tr><td>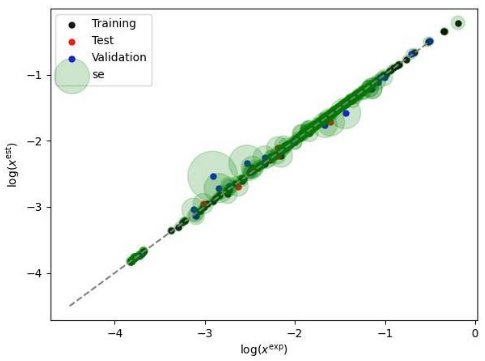
</td><td>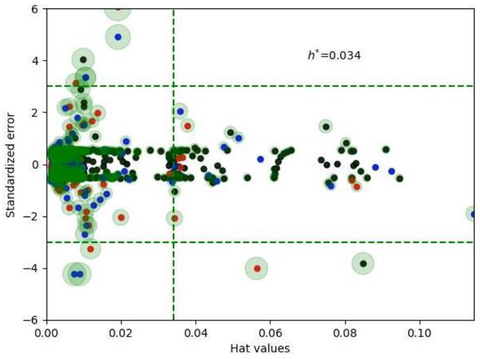
</td></tr>
<tr><th colspan="2">c</th></tr>
</table>

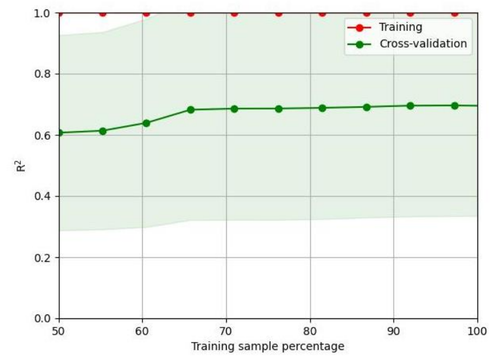


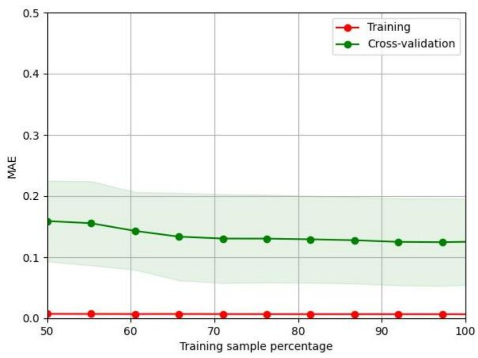


**CatBoostRegressor**

| a | b |
|---|---|

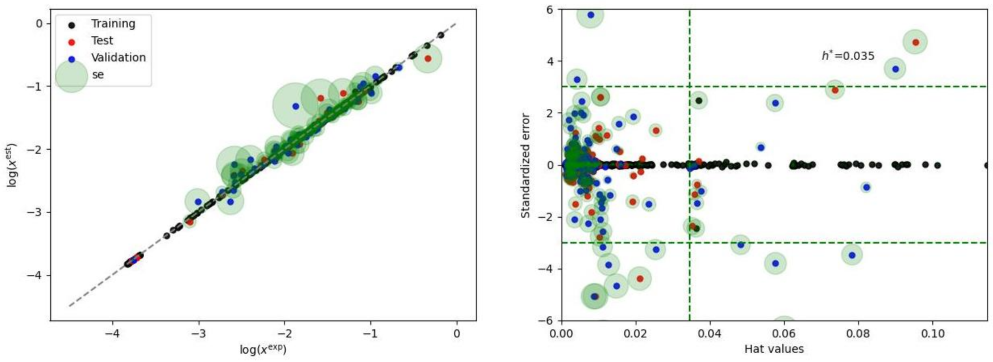


c

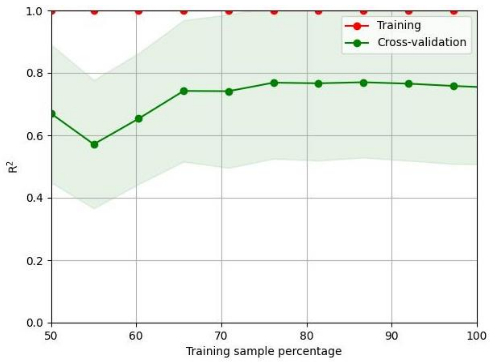


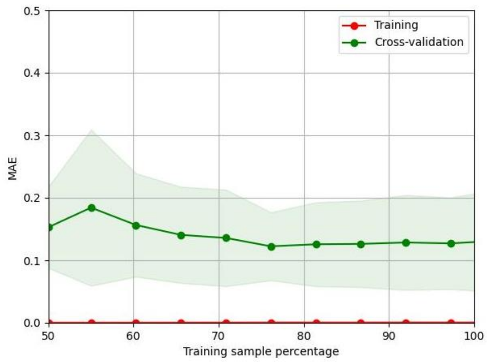


**XGBRegressor**

| a | b |
|---|---|

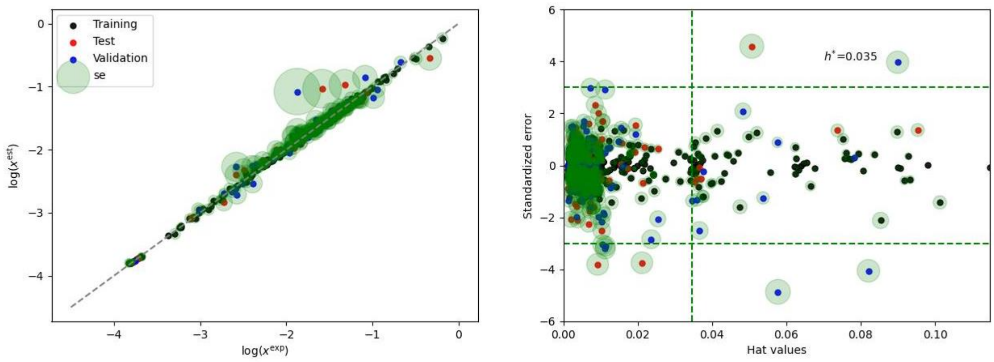


c

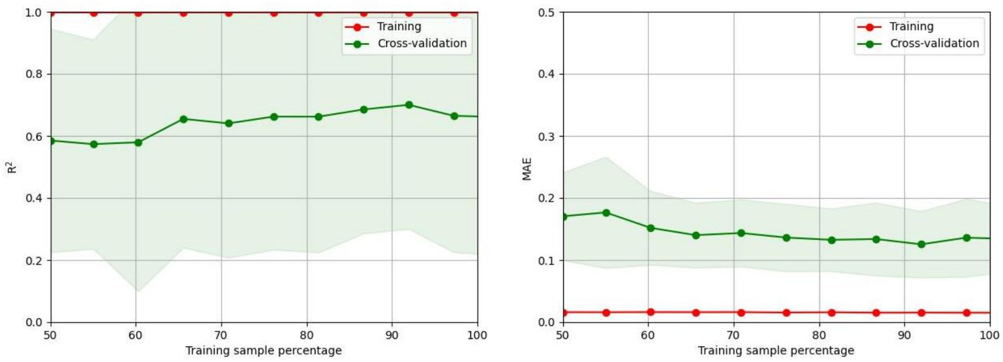


**HistGradientBoostingRegressor**

**a**

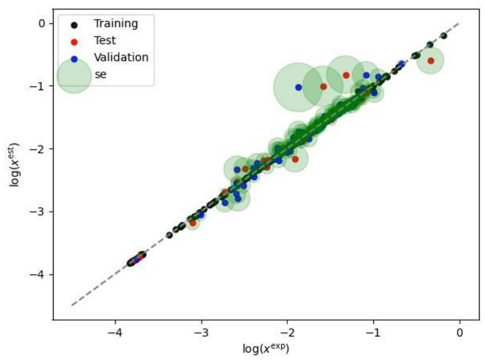


**b**

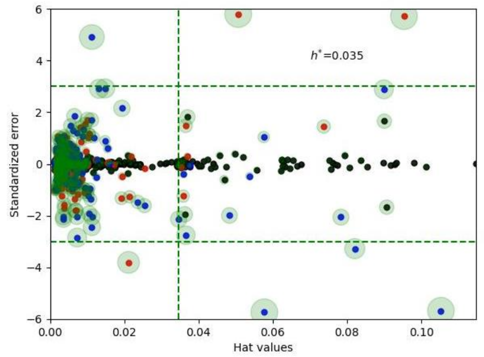


**c**

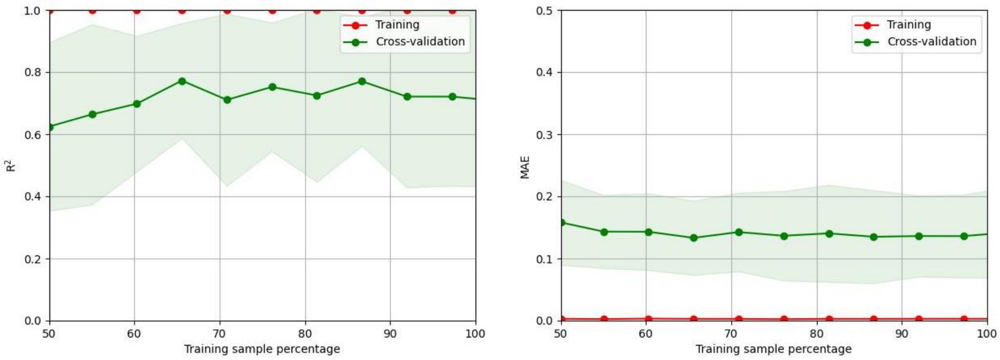

***Table S6. Graphical illustration of the of the regression model performance belonging to set B. The panels a,b, and c document the correlation between computed and consensus solubility values with annotation of the standard deviation as circles radius, applicability domain plots, and results of learning curve analysis concerning both $R^2$ and MAE, respectively.***

<table>
<tr><th colspan="2">GaussianProcessRegressor</th></tr>
<tr><th>a</th><th>b</th></tr>
<tr><td>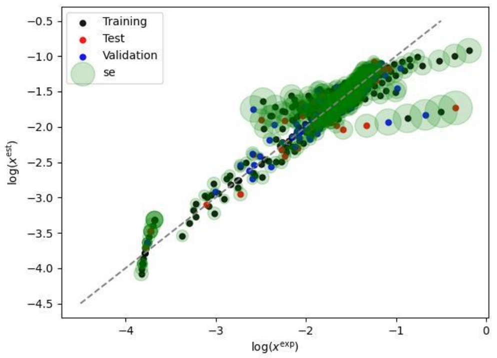
</td><td>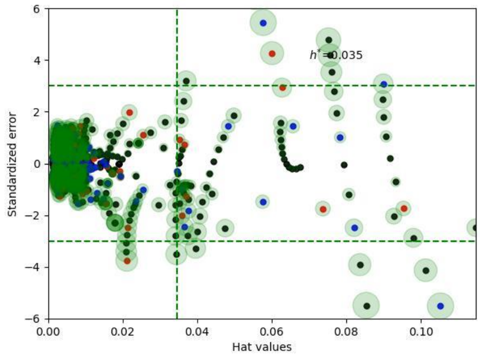
</td></tr>
<tr><th colspan="2">c</th></tr>
<tr><td colspan="2">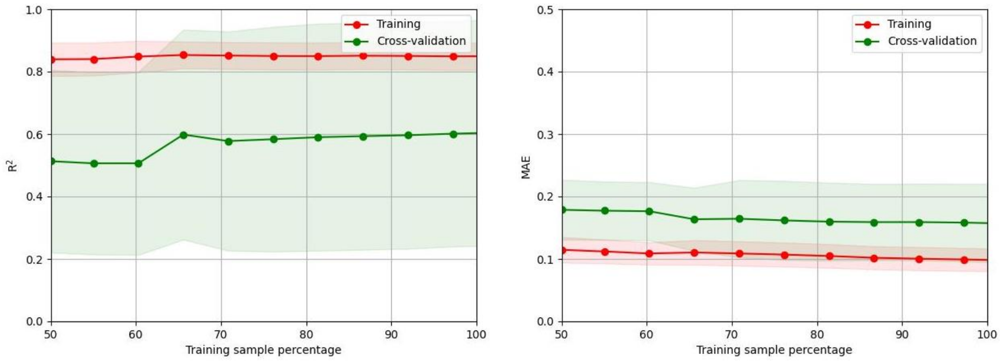
</td></tr>
</table>

<table>
<tr><th colspan="2">BaggingRegressor</th></tr>
<tr><th>a</th><th>b</th></tr>
<tr><td>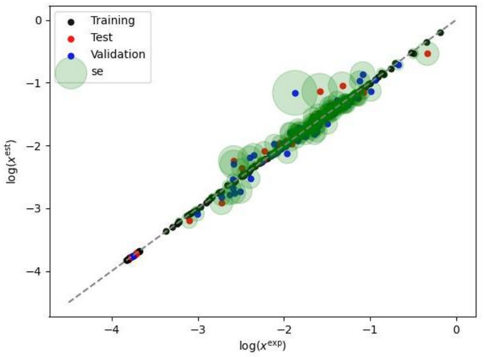
</td><td>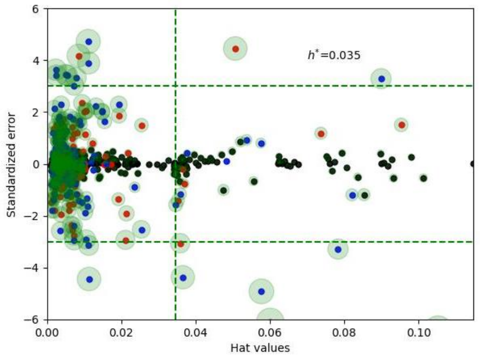
</td></tr>
<tr><th colspan="2">c</th></tr>
</table>

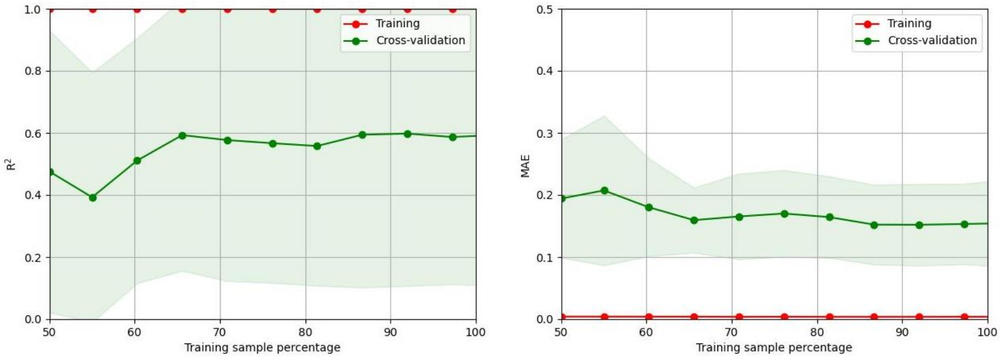


| RandomForestRegressor | |
|---|---|
| **a** | **b** |

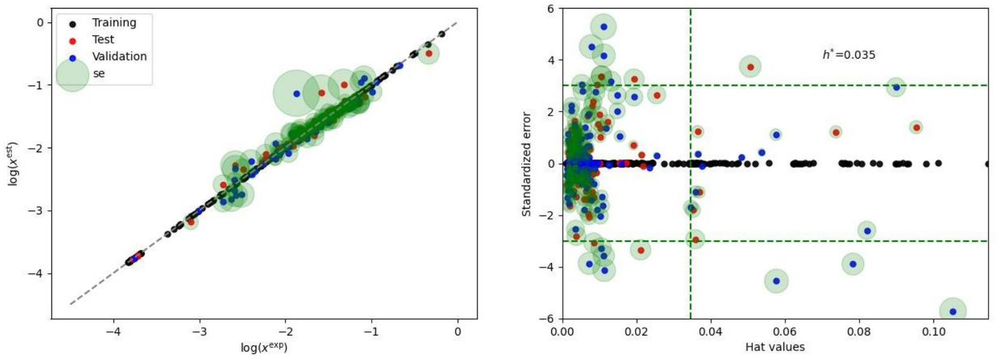


**c**

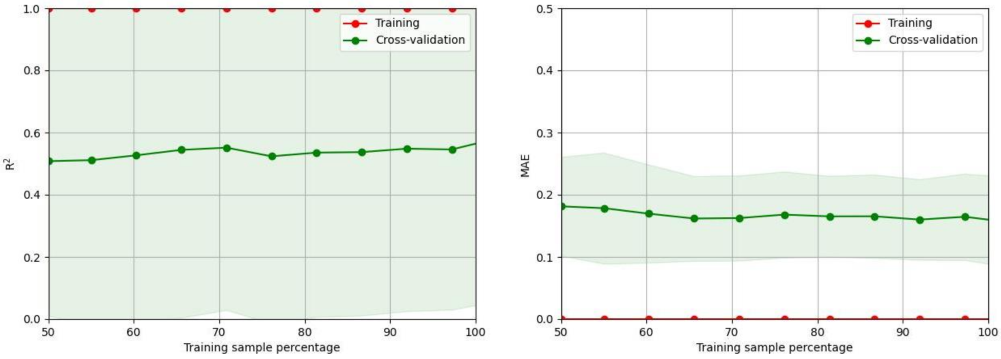


| LGBMRegressor | |
|---|---|
| **a** | **b** |

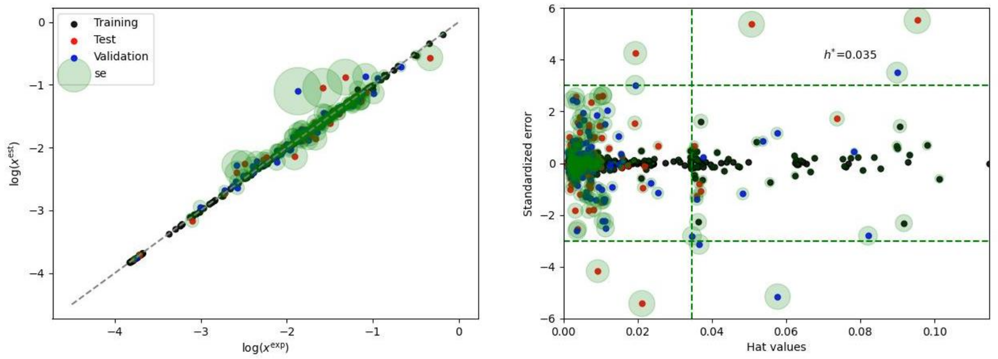


**c**

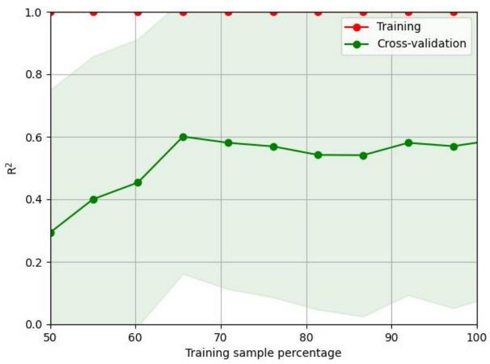

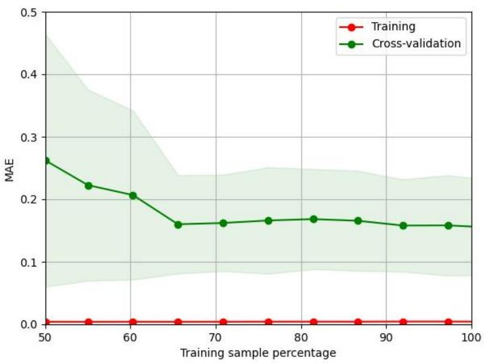


| MLPRegressor | |
|---|---|
| **a** | **b** |

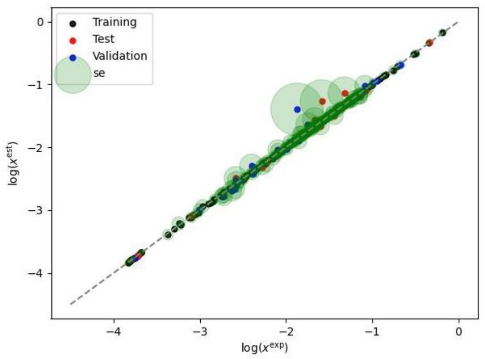

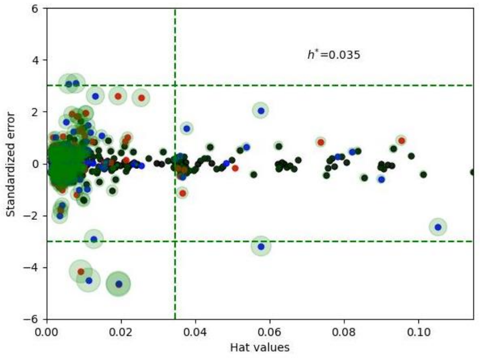


**c**

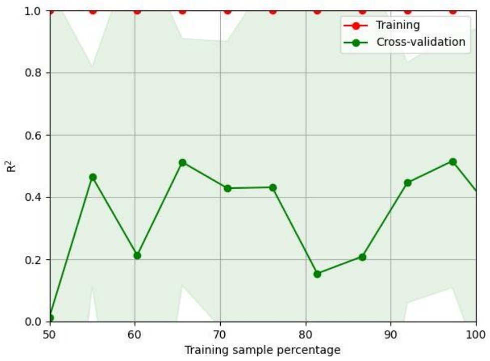

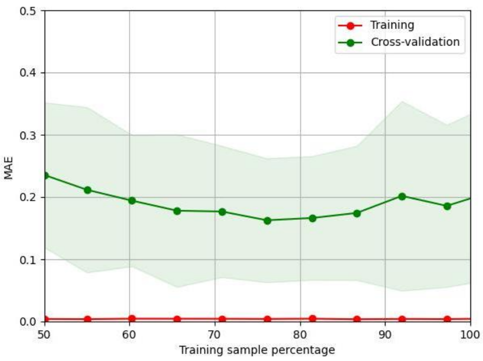


| LassoLars | |
|---|---|
| **a** | **b** |

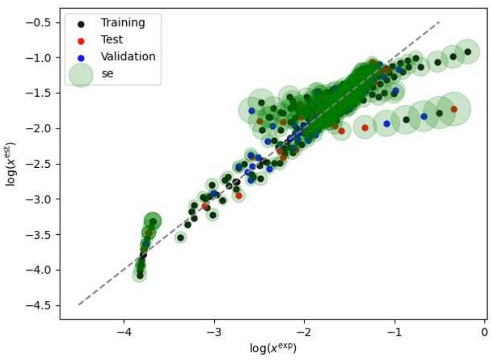

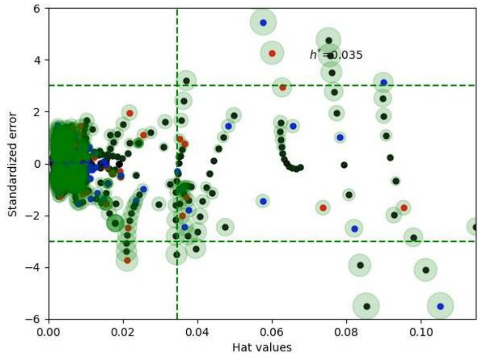


**c**

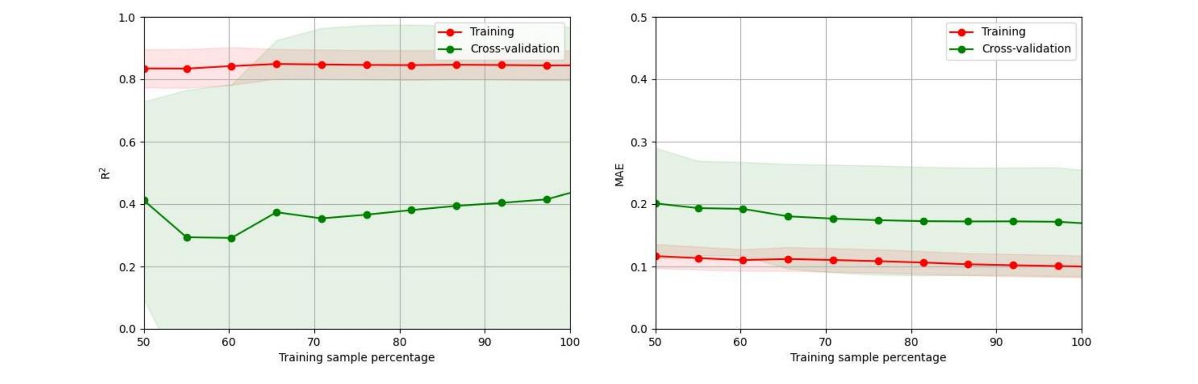


**LassoLarsCV**

| a | b |
|---|---|

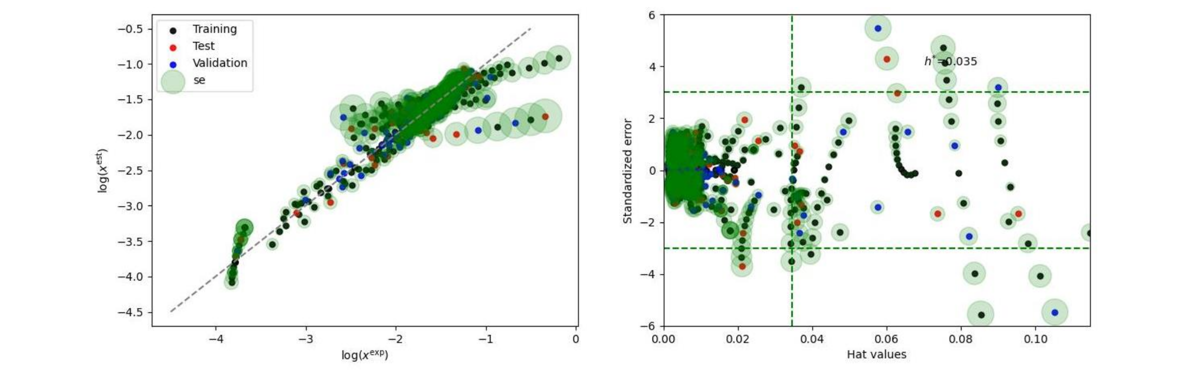


**c**

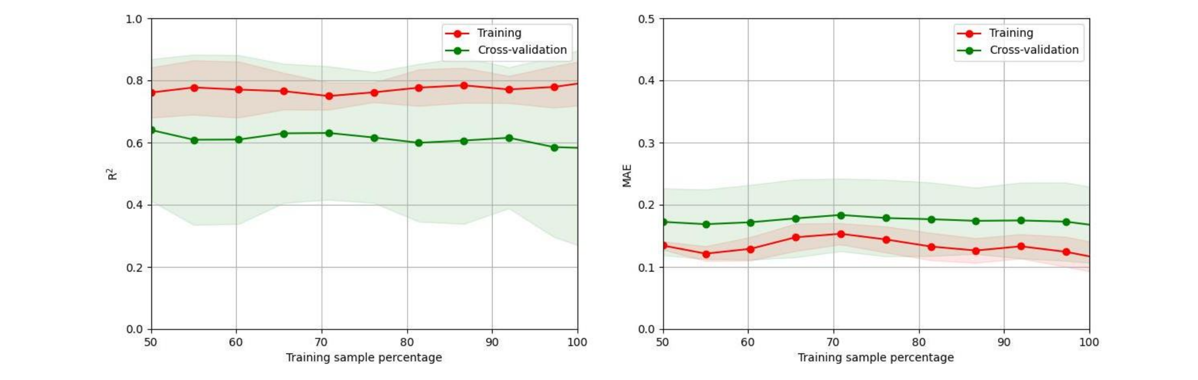


**Ridge**

| a | b |
|---|---|

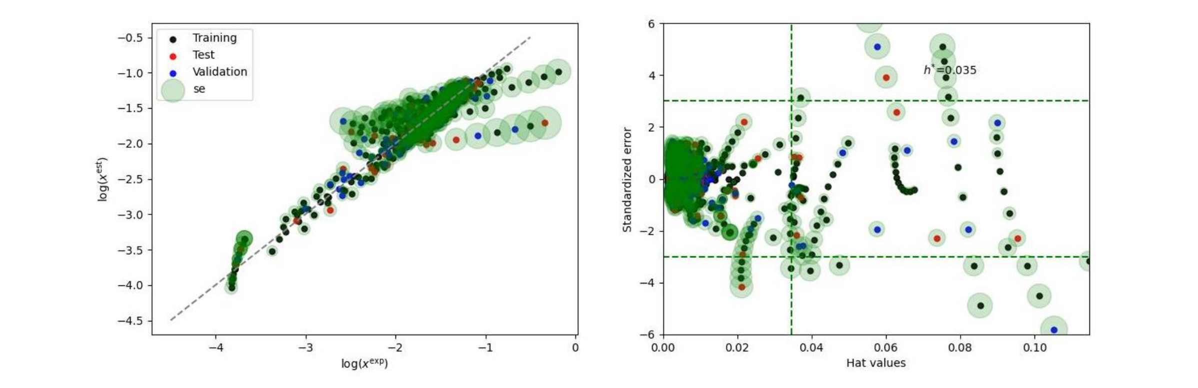


**c**

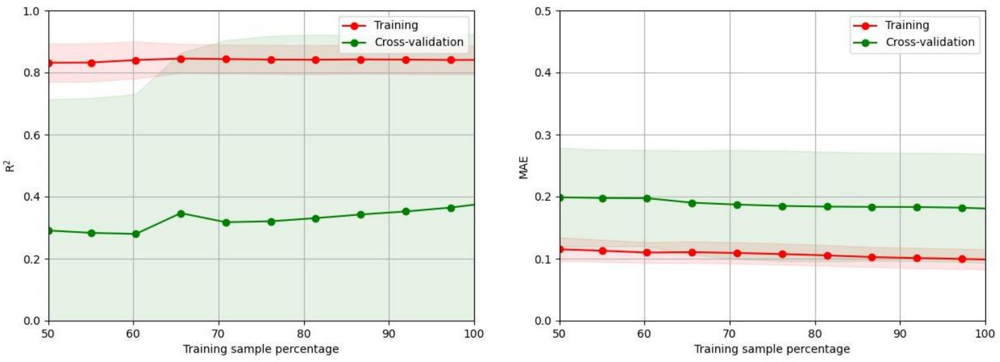


**KNeighborsRegressor**

**a** **b**

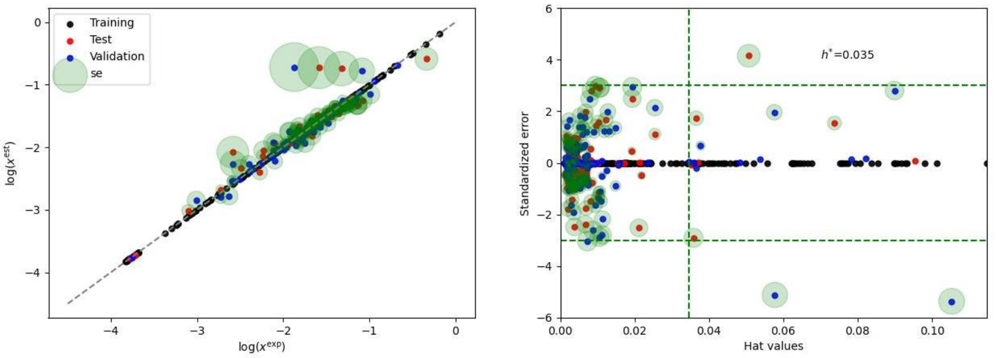


**c**

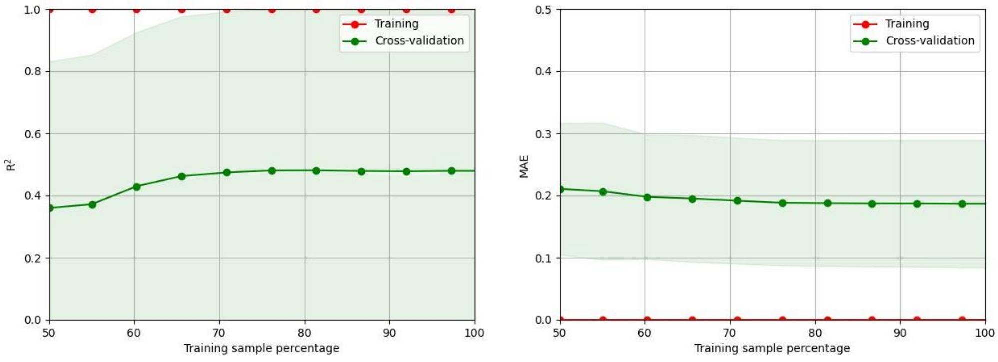


**AdaBoostRegressor**

**a** **b**

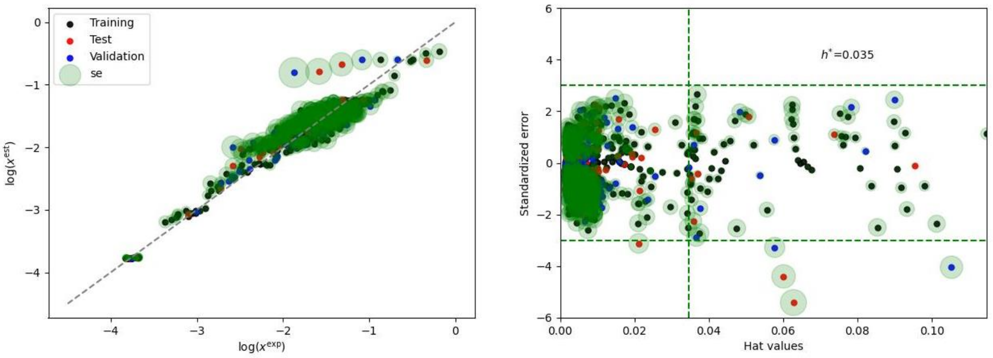


**c**

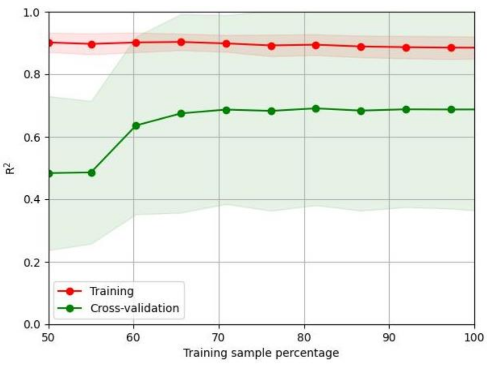


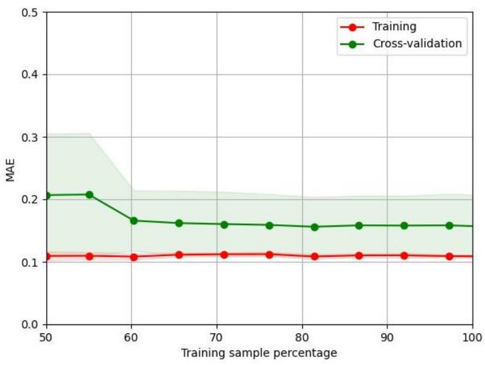


**OrthogonalMatchingPursuitCV**

a b

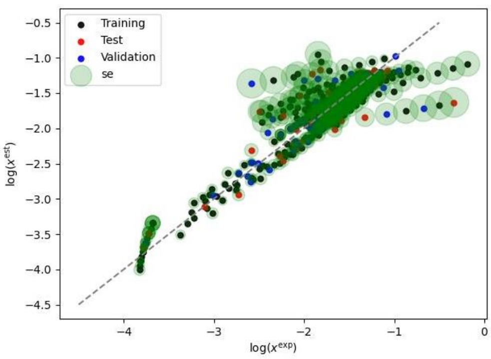


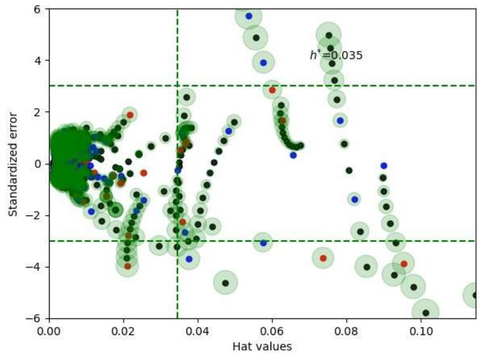


c

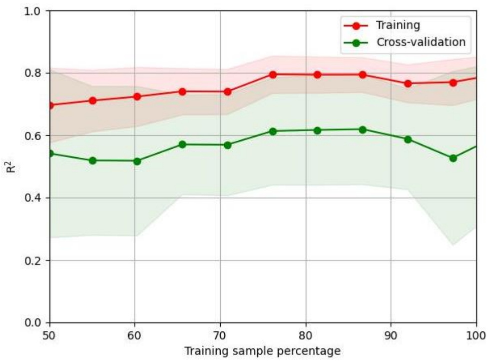


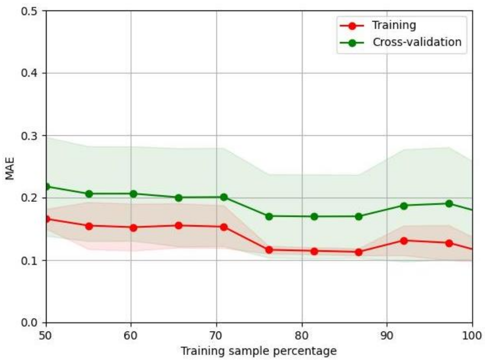


**TransformedTargetRegressor**

a b

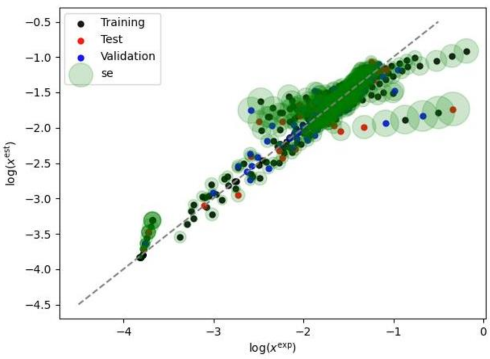


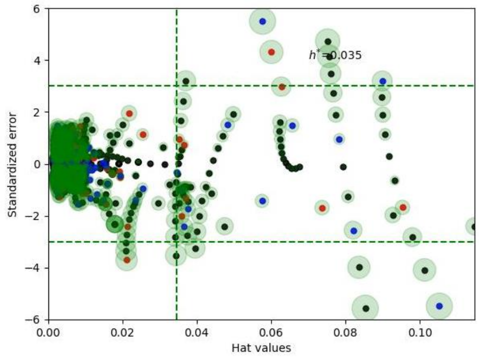


c

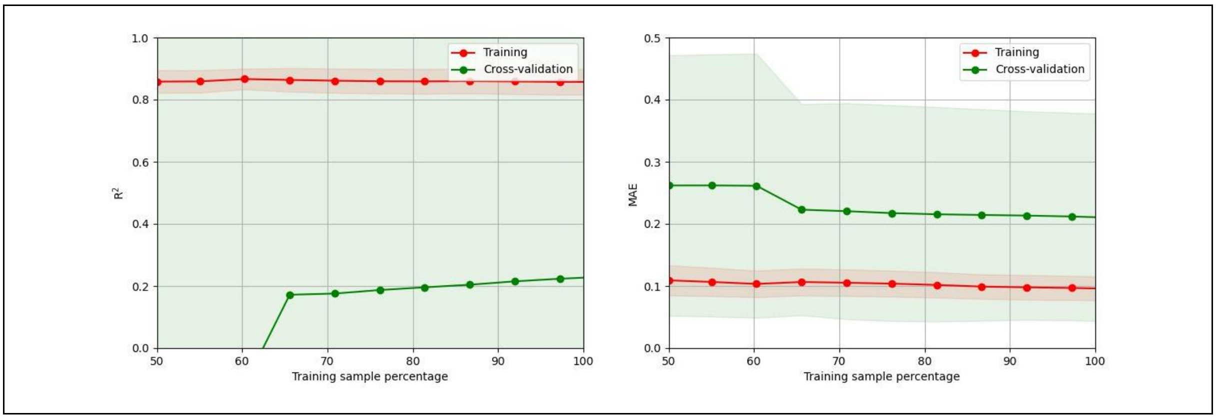

Training
Cross-validation
R²
Training sample percentage
MAE
